# Thermally Drawn Flexible Fiber Sensors: Principles, Materials, Structures, and Applications

**DOI:** 10.1007/s40820-025-01840-y

**Published:** 2025-07-18

**Authors:** ZhaoLun Zhang, Yuchang Xue, Pengyu Zhang, Xiao Yang, Xishun Wang, Chunyang Wang, Haisheng Chen, Xinghua Zheng, Xin Yin, Ting Zhang

**Affiliations:** 1 Institute of Engineering Thermophysics, Chinese Academy of Sciences, Beijing, 100190 People’s Republic of China; 2 Nanjing Institute of Future Energy System, Nanjing, 211135 People’s Republic of China; 3 University of Chinese Academy of Sciences, Beijing, 100049 People’s Republic of China; 4 Key Laboratory of Long-Duration and Large-Scale Energy Storage, Chinese Academy of Sciences, Beijing, 100190 People’s Republic of China; 5 Department of Orthopedics, Capital Center For Children’s Health, Capital Medical University, Beijing, 100020 People’s Republic of China; 6 Department of Orthopedics, Fourth Medical Center of PLA General Hospital, Beijing, 100048 People’s Republic of China; 7 University of Chinese Academy of Sciences, Nanjing, 211135 People’s Republic of China; 8 Innovation Academy for Light-Duty Gas Turbine, Chinese Academy of Sciences, Beijing, 100190 People’s Republic of China

**Keywords:** Thermally drawn fiber sensors, Sensing principles, Temperature sensors, Mechanical sensors, Multifunctional sensors

## Abstract

Flexible fiber sensors, with their excellent wearability and biocompatibility, are essential components of flexible electronics. However, traditional methods face challenges in fabricating low-cost, large-scale fiber sensors. In recent years, the thermal drawing process has rapidly advanced, offering a novel approach to flexible fiber sensors. Through the preform-to-fiber manufacturing technique, a variety of fiber sensors with complex functionalities spanning from the nanoscale to kilometer scale can be automated in a short time. Examples include temperature, acoustic, mechanical, chemical, biological, optoelectronic, and multifunctional sensors, which operate on diverse sensing principles such as resistance, capacitance, piezoelectricity, triboelectricity, photoelectricity, and thermoelectricity. This review outlines the principles of the thermal drawing process and provides a detailed overview of the latest advancements in various thermally drawn fiber sensors. Finally, the future developments of thermally drawn fiber sensors are discussed.

## Highlights

- The review briefly introduces the principle, material selection criteria, and development of the thermal drawing process.
- Based on different stimuli, the review comprehensively summarizes the latest progress in thermally drawn temperature, acoustic, mechanical, chemical, biological, optoelectronic, and multifunctional sensors.
- The review discusses the future development trends of thermally drawn fiber sensors in terms of material, structure, fabrication, function, and stability.

## Graphical abstract

**Figure:**
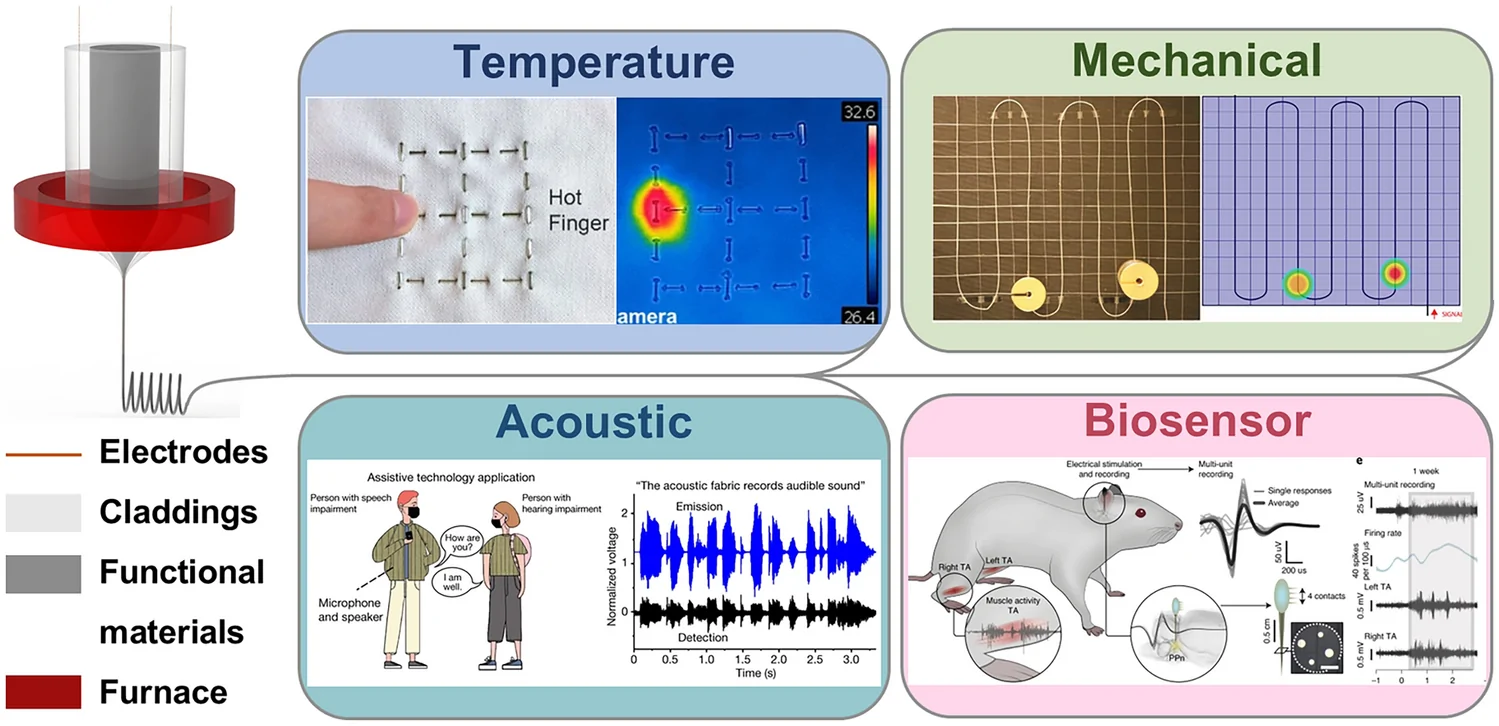

## Introduction

In recent decades, flexible electronics have advanced rapidly. Due to their bendable and stretchable properties, flexible electronic products have been widely applied in biomedicine, intelligent robotics, environment, and health monitoring [1–10], profoundly transforming people's lives. As a key component of flexible electronics, sensors act as a bridge connecting humans with the external environment. They can convert external stimuli into real-time electrical signals (such as capacitance and resistance) with high reliability and sensitivity. Their application demands are increasing significantly [11–14]. In the early research on flexible sensors, most sensing elements were fabricated by combining inorganic rigid materials (such as Si and Au) with flexible substrates [15–17]. However, these partially flexible sensors were difficult to apply to complex surfaces such as human skin and robots. As a result, fully flexible sensors based on flexible and stretchable materials like organic materials and metal nanowires have emerged rapidly [18, 19]. Depending on the fabrication method, flexible sensors can be classified into film and fiber-based sensors [20, 21]. Compared to film, fiber, as the smallest unit in the textile industry, offers better breathability and wearing comfort while maintaining excellent flexibility and biocompatibility, making it more advantageous for applications in smart textiles and wearable devices [22–24].

From natural fibers to synthetic fibers and further to revolutionary carbon fibers and optical fibers, multi-functionalization has always been the development trend of fibers [25–28]. To date, various methods such as dry spinning [29], wet spinning [30], melt spinning [31], microfluidic spinning [32], electrospinning [33, 34], physical vapor deposition (PVD) [35], chemical vapor deposition (CVD) [36], and 3D printing [37] have been applied to fabricate multifunctional fiber sensors. However, they all have some insurmountable disadvantages. Spinning processes often involve multiple processing steps and prolonged overall fabrication times [38]. Moreover, microfluidic spinning process is more complex in terms of its process [32]. PVD and CVD typically require harsh conditions such as high-temperature and high-vacuum environment [39]. 3D printing of fibers is constrained by limited printable lengths and inherently slow deposition rates [38]. Among these processes, thermal drawing process stands out as a superior technology: it obviates the need for complex environment, enables customization of diverse fiber architectures, and supports functional integration across length scales spanning from nanometers to kilometers [39]. This technique represents the most promising pathway for low-cost, large-scale production of functional fibers, with exceptional industrial applicability. Furthermore, this process has excellent process compatibility. Its combination with various technologies such as 3D printing, direct imprinting, and photolithography can further promote the high-performance and multifunctional applications of thermally drawn fiber sensors (TDFSs) [40–42].

**Fig. 1:**
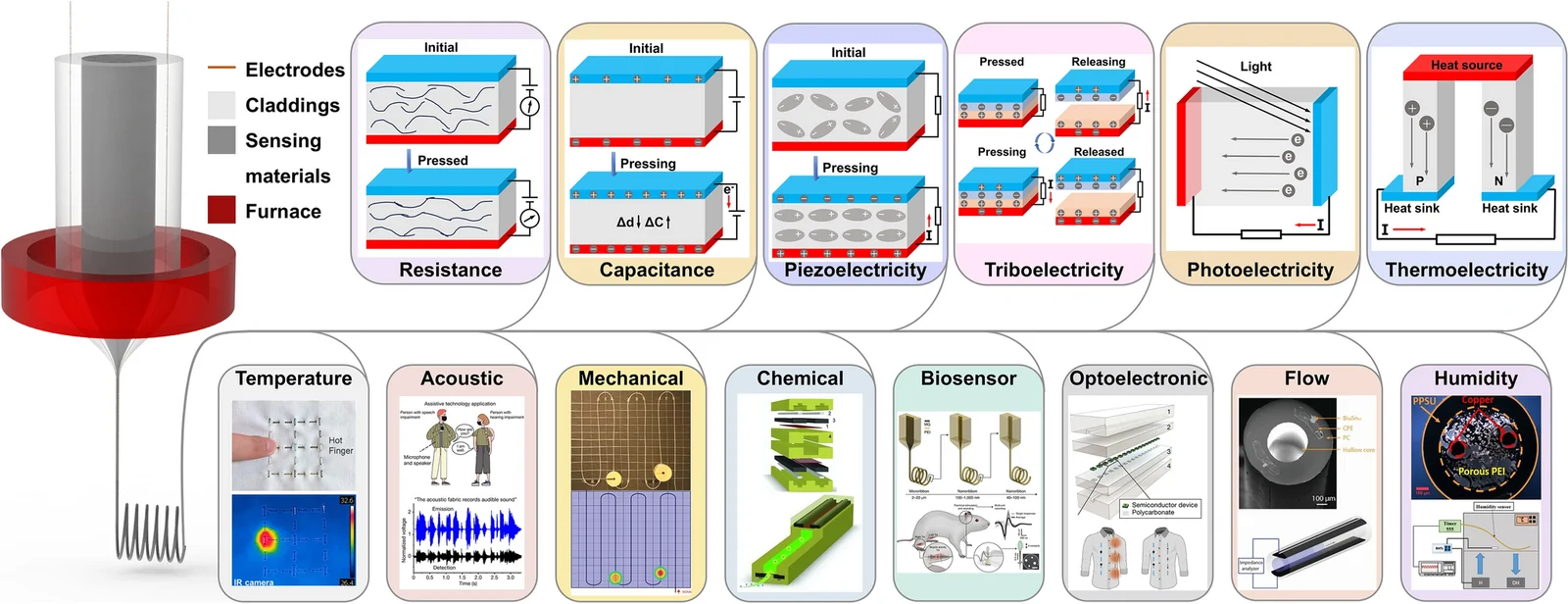
Sensing principles and applications of thermally drawn fiber sensors. Sensing principles: resistance, capacitance, piezoelectricity, triboelectricity, photoelectricity, and thermoelectricity. Applications: Temperature sensor. Reproduced with permission [48] Copyright 2019 American Chemical Society. Mechanical sensor. Reproduced with permission [49] Copyright 2020 Wiley. Acoustic sensor. Reproduced with permission [19] Copyright 2022 Springer. Chemical sensor. Reproduced with permission [50] Copyright 2012 Wiley. Biosensor. Reproduced with permission [51] Copyright 2022 Springer. Optoelectronic sensor. Reproduced with permission [43] Copyright 2018 Springer. Flow sensor. Reproduced with permission [52] Copyright 2019 Wiley. Humidity sensor. Reproduced with permission [53] Copyright 2019 Multidisciplinary Digital Publishing Institute

The third revolution of science and technology is driving the rapid development of the Internet of Things (IoT) and flexible electronics. It is urgent to increase the integration of flexible sensors and simplify wearable device structures [12]. Recently, Moore's law for thermally drawn fibers has been proposed, indicating the continuous high integration of materials and devices in TDFSs [43], giving a powerful boost to the advancement of flexible sensors. Although a few years ago, previous reviews have summarized the thermal drawing process and its diverse applications [44–47]. TDFSs have undergone substantial expansion in both research domains and novel applications in recent years. There is a pressing need to systematically summarize their latest advancements, analyze future development tendency, and provide a consolidated reference for researchers working in this rapidly evolving field. Therefore, this article will introduce the mechanism and development trend of the thermal drawing process, review common sensing principles, and provide a detailed discussion of the applications of TDFSs in temperature, mechanical, acoustic, chemical, biological, optoelectronic, flow, and humidity sensing (Fig. 1). Finally, the future development directions of TDFSs are discussed. This review aims to guide the development of flexible fiber sensors.

## Thermal Drawing Process

The thermal drawing process is an emerging fiber fabrication method initially used to fabricate optical fibers [54, 55]. Due to its capability to control fiber structures and sizes, it has garnered significant attention in materials science and textiles. Compared to traditional fiber fabrication methods, the thermal drawing process enables the rapid, large-scale, and automated production of multimaterial fibers incorporating high-density devices and diverse functionalities. This technology aligns well with the requirements of textile manufacturing processes, providing a universal platform for the scalable production of smart textiles.

### Mechanism of the Thermal Drawing Process

The mechanism of the thermal drawing process is to integrate fiber formation and functionalization into a continuous drawing process. During this process, all components (including claddings, electrodes, functional materials, etc.) are co-drawn in a viscous molten state [56]. The first step in the specific process is to fabricate a macroscopic preform. The preform, which serves as an enlarged model of the fiber, is the crucial component of the thermal drawing process. Various methods can be used to fabricate preforms, such as rod-in-tube [57], hot pressing [58], stack-and-draw [59], thermal consolidation [60], film rolling [61], 3D printing [62], double-crucible [63], and melt casting [64]. The preform is then mounted in a drawing tower, passing sequentially through the preheating zone, heating zone, and insulation zone from top to bottom. It is gradually heated to 50–100 °C above the cladding's glass transition temperature (*T*_g_) or melting temperature (*T*_m_). After maintaining this temperature for a period, the preform undergoes necking. At this stage, the fiber is drawn under external force, enabling continuous and controllable production (Fig. 1). The resulting fiber retains the same composition and structure as the preform but with a diameter reduced to the submillimeter scale, integrating multiple materials and functionalities.

The diameter of the fiber can be adjusted by thermal drawing parameters, including the drawing temperature (*T*_D_), the fiber drawing speed (*v*_d_), and the preform feeding speed (*v*_f_). According to the law of volume conservation, the fiber diameter (*D*_fiber_) can be expressed as:

1 $${\mathrm{D}}_{\mathrm{fiber}} = {\mathrm{D}}_{\mathrm{preform}}\times \sqrt{\frac{{\mathrm{v}}_{\mathrm{f}}}{{\mathrm{v}}_{\mathrm{d}}}}$$

The above formula is merely a simple reference for controlling fiber diameter. It is important to note that thermal drawing is a complex process, and various factors such as material viscosity, thermal expansion coefficient, chemical stability, and surface tension [65] can also influence it. These factors are not accounted for in the formula. Additionally, to increase the drawing stress for stabilizing the fiber dimensions, it is often necessary to reduce the temperature [66].

### Material Selection Criteria

Although the thermal drawing process theoretically enables the co-drawing of multiple materials, differences in the thermal, mechanical, and chemical properties of various materials can affect the uniformity of fiber structure and cross-sectional integrity along the fiber length. Therefore, certain criteria must be followed when selecting materials.

Firstly, all co-drawn materials should have similar *T*_g_ or *T*_m_, and the drawing temperature must be higher than the *T*_g_ or *T*_m_ but lower than the boiling temperature [67]. Secondly, to prevent fractures and cracks, materials should also exhibit similar thermal properties, such as Vicat softening temperature (VST) [68], thermal expansion coefficients (*α*), and viscosities. This helps control residual stresses caused by thermal–mechanical mismatch in fiber [69]. Table 1 summarizes the *T*_g_, VST, and* α* of common polymers. Additionally, materials should demonstrate good adhesion and wettability in both viscous and solid states to avoid cracking after rapid thermal cooling and quenching [70]. Furthermore, since the cladding bears most of the stress, the process must occur within a relatively high-viscosity range, typically between 10^4^ and 10^8^ Pa s [56]. It is noteworthy that viscosity exhibits significant temperature dependence, and determining whether the viscosity of specific materials matches requirements typically necessitates analysis with rheometer. Ideal cladding should be amorphous. In addition to conventional glass materials, thermoplastic materials can also serve as claddings for TDFSs. For instance, highly transparent materials such as polymethyl methacrylate (PMMA) and polycarbonate (PC) find extensive applications in the field of biosensing [71]. Meanwhile, thermoplastic elastomers (TPEs) that meet rheological criteria like SEBS play an indispensable role in acoustic and mechanical sensing due to their unique properties [19, 72, 73]. Notably, the thermal drawing process induces molecular chain realignment in polymers. Taking P(VDF-TrFE) as an example, spontaneous α-phase to β-phase transition occurs during this process, leading to altered material properties. This transformation mechanism also necessitates consideration in material selection. Finally, potential chemical diffusion and reactions must also be considered [56].

**Table 1:** *T*_g_, VST, and* α* of common polymers

Materials	*T*_g_ (°C)	VST (°C)	*α* (10^–6^ m/(m °C))
PC	139–150	123–157	65–70
PMMA	100–120	87–125	50–90
SEBS	− 90	~ 165	61–65
LDPE	− 110	90–125	108–200
PVDF	− 42 to − 25	~ 136	128–140
PSU	187–190	175–185	55–60
PEI	217–220	210–226	~ 100
TPU	− 49 to − 12	66–145	~ 57
PS	63–112	~ 120	~ 70

### Development Trend of the Thermal Drawing Process

Over decades of development in the thermal drawing process, three types of thermally drawn fibers have emerged based on differences in structure and fabrication processes. In 2002, the first generation of thermally drawn fiber was fabricated by Yoel Fink’s group at the Massachusetts Institute of Technology (MIT), representing the initial structure and fabrication process [54, 55]. These fibers were directly drawn from preforms without post-processing, maintaining continuous and uniform composition across their entire length. During the thermal drawing process, only physical contraction of the preform occurred, and it was impossible to adjust or customize the structure along the fiber length. In 2012, the second generation of thermally drawn fiber was fabricated, incorporating post-processing steps into the first generation [74]. For example, during a post-processing step involving thermal perturbation that causes capillary rupture, the cylindrical fibers evolve toward more stable spherical shapes due to the Rayleigh instability phenomenon when subjected to thermal disturbances [75]. In 2018, the third generation of thermally drawn fibers emerged. These fibers featured pre-designed empty slots for electrodes or microdevices within the preform, enabling the gradual establishment of electrical contacts during the drawing process [43]. Based on this advancement, fibers containing a series of microdevices were fabricated [43, 76, 77]. Concurrently, Yoel Fink’s group proposed the concept of "Moore’s law for fibers" [43]. Analogous to Moore’s law in microelectronics, this principle predicts the continuous high-density integration of functional materials and devices into fibers and textiles, paving the way for smart textiles with broader applications and even computationally predictable functionalities. In the future, thermally drawn fibers will evolve toward more diverse material selections, complex structures, and highly integrated functionalities. They will also become more closely integrated with artificial intelligence and machine learning. Smart textiles based on thermally drawn fibers will further transcend the limitations of conventional products, evolving into sophisticated computational platforms to serve human life.

## Applications of Thermally Drawn Fiber Sensors

In recent years, thermally drawn fiber sensors (TDFSs) have developed rapidly. Various sensors have been fabricated based on principles such as resistance, capacitance, piezoelectricity, triboelectricity, photoelectricity, and thermoelectricity. These sensors cover multiple functions, including temperature, acoustic, mechanical, chemical, biological, optoelectronic, flow, and humidity sensing. Table 2 summarizes the principles, functional materials, claddings, and electrodes of the eight main types of sensors. This chapter introduced the latest advancements in each field according to different sensor types.

**Table 2:** Summary of the principles, functional materials, claddings, and electrodes for eight types of sensors

Sensor types	Principles	Functional materials	Claddings	Electrodes	References
Temperature sensors	Resistance	rGO/PLA	PS	rGO/PLA	[78]
	Thermoelectricity	SEBS	SEBS	Dual GaInSn	[79]
	Capacitance	LDPE	LDPE	Copper wire	[49]
Acoustic sensors	Piezoelectricity	P(VDF-TrFE)/BaTiO_3_	SEBS	CPE and copper wire	[19]
Mechanical sensors	Resistance	CPE	SEBS	CPE	[80]
	Capacitance	Galinstan	SEBS	Galinstan	[72]
	Piezoelectricity	BTO-PVDF	PC	C-LDPE	[81]
	TENG	PVDF	PVDF	CPE	[41]
	Photoelectricity	STPU	STPU	–	[82]
Chemical sensors	Photoelectricity	Se_97_S_3_	PSU	Sn_63_Pb_37_	[50]
	Electrochemistry	CPC and Pt-MG	PEI	CPC and Pt-MG	[83]
Biosensors	Photoelectricity	PC/PMMA	PDMS	CNT sheets	[84]
	Capacitance	COC	PC	CPE with 2% CNF	[85]
	TENG	PVDF	PSU	Copper tape	[86]
Optoelectronic sensors	Photoelectricity	Ge	PC	Copper wire	[87]
Flow sensors	Capacitance	Fluid in the microfluidic channel	PC	CPE and Bi_58_Sn_42_	[52]
Humidity sensors	Capacitance	Porous PEI	PPSU	Copper wire	[53]

### Temperature Sensors

Temperature sensors are among the most widely used sensors in military, medical, biological, and industrial fields. Monitoring temperature can reveal a wealth of chemical, physical, and biological information, which is helpful for practical applications. The principles of temperature sensors mainly include thermistor, thermoelectric effect, capacitance, and infrared imaging [48, 88, 89]. Among these, infrared imaging has difficulty monitoring irregular surfaces' temperature. Besides, it is limited by space and relatively expensive [90]. Therefore, it is generally not used in flexible temperature sensors. Temperature sensors based on thermistors convert temperature changes into resistance changes. Their principle is based on the following formula:

2 $$\mathrm{R}=\rho\frac{\mathrm{L}}{{\mathrm{A}}}$$

**Fig. 2:**
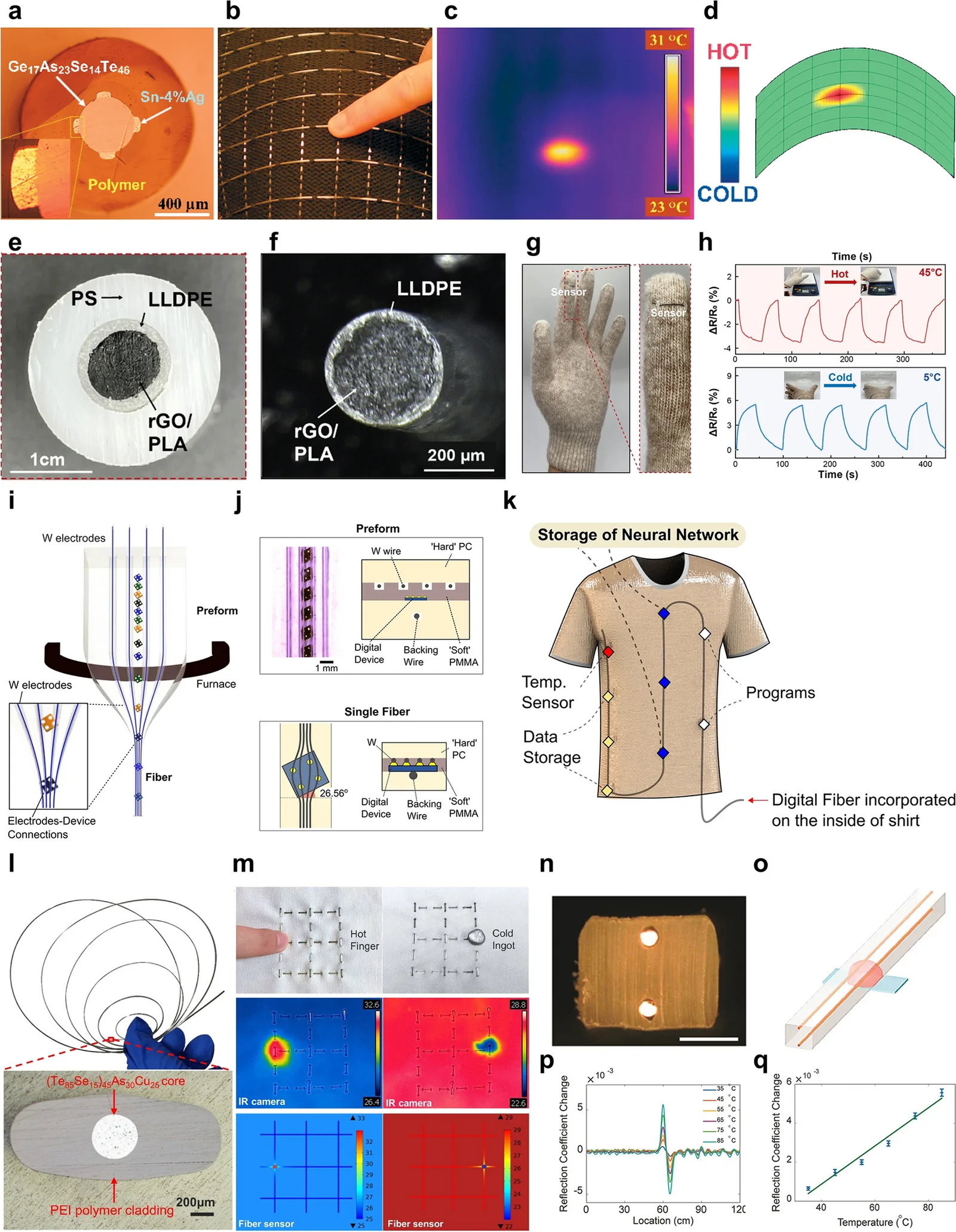
Thermally drawn fibers for temperature sensing. **a** A Micrograph of the cross section of a thermistor-based fiber sensor. **b** Fiber array for spatial sensing. **c**, **d** Infrared radiographs and thermal maps of fiber array. Reproduced with permission [93] Copyright 2006 Wiley. **e**, **f** Structure of the perform and fiber with rGO/PLA thermistor in the core. **g**, **h** Image of the TDFS sewn into the top of a glove and temperature test with multiple touches. Reproduced with permission [78] Copyright 2023 Springer. **i**, **j** TDFS with integrated multiple microelectronic components. **k** Shirt integrated with fibers. Reproduced with permission [77] Copyright 2021 Springer. **l** Fiber structure with TE material in the core. **m** Temperature sensor network based on 3 × 3 TE fiber arrays for simultaneous detection and localization of heat source. Reproduced with permission [48] Copyright 2019 American Chemical Society. **n** Fiber structure of the FDR-based TDFS. **o** Image of the fiber being heated at one location. **p**, **q** Temperature response and reflection coefficient change of the fiber. Reproduced with permission [49] Copyright 2020 Wiley

In the formula, *ρ*, *L*, and *A* denote the resistivity, length, and cross-sectional area of the sensing material, respectively. Generally, the resistance change mainly consists of two parts: the geometry change and the resistivity change [91]. In some semiconductors, the effect of temperature on resistivity constitutes the main part of the resistance change. The temperature coefficient of resistance (TCR), which represents the ratio of resistance change to temperature change, is an indicator of thermistor performance [92]. Chalcogenide semiconductors are commonly used in temperature sensors [93]. Mehmet et al. [93] firstly fabricated a temperature sensor using the chalcogenide semiconductor Ge_17_As_23_Se_14_Te_46_ (GAST) as the thermistor via the thermal drawing process. They selected 96%Sn–4%Ag alloy electrodes and polysulfone (PSU) polymer cladding. The final fiber is shown in Fig. 2a. The electrical signal of a single fiber was proportional to the integral of thermal excitation over its entire length. Fiber maintained good ohmic characteristics at both low (11 °C) and high temperatures (58 °C). To measure the spatial temperature, an 8 × 8 fiber array with 1 cm spacing was further fabricated (Fig. 2b) and woven into a fabric, capable of precisely locating heat sources and quantifying temperature (Fig. 2c, d). The high modulus of semiconductors and metal electrodes limits the application of fibers in wearable devices, while the TCR of polymers is not as significant as that of semiconductors. Graphene, with its excellent electrical properties and unique temperature response characteristics, has been widely used in temperature sensors [94, 95]. Adding it into polymers can enhance the TCR while maintaining flexibility. Ryu et al. [78] fabricated a TDFS using reduced graphene oxide (rGO) and polylactic acid (PLA) as the thermistor. The outer layers consisted of linear low-density polyethylene (LLDPE) and a sacrificial polystyrene (PS) cladding, as shown in Fig. 2e, f. When woven into a glove, it could detect touch temperature (Fig. 2g, h). Moreover, the fiber exhibited consistent and reliable temperature responses under repeated mechanical stress and chemical exposure, meeting the requirements for long-term applications in flexible wearable fiber sensors.

Different from traditional thermistor-based temperature sensors, Loke et al. [77] integrated hundreds of dispersed thermistors and storage elements into the interior of a thermally drawn fiber, as shown in Fig. 2i, j. A single node at the end of the fiber can independently access multiple devices inside the fiber. A single fiber achieves the functions of temperature monitoring and storage simultaneously, overcoming the previous limitation of relying on parallel connections of multiple devices. When woven into a shirt (Fig. 2k), the fiber could monitor body temperature under various human motions and continuously store temperature data for several days. Furthermore, through a neural network, by analyzing the unknown temperature–time data, the type of physical activity could be inferred with an accuracy of 96%. This fiber can perform different functions at different positions, reducing the number of devices required for the same function and improving the integration level of flexible electronics.

The thermoelectric (TE) effect refers to the energy conversion between heat and electricity, including the Seebeck effect, Peltier effect, and Thomson effect. Temperature sensors based on the thermoelectric effect mainly utilize the Seebeck effect, which means that a temperature gradient creates a potential difference between the contact surfaces of different materials. In a closed circuit, this potential difference will generate an electric current, thereby converting thermal energy into electrical energy [96]. The potential difference *V* can be expressed by the following formula:

3 $$\mathrm{V} = \mathrm{S}\Delta\mathrm{T}$$

In the formula, *S* is the relative Seebeck coefficient, and Δ*T* is the temperature difference. Therefore, materials with high Seebeck coefficients are crucial for high-sensitivity and high-resolution temperature measurements [97]. The thermoelectric performance of amorphous semiconductor glass ((Te_85_Se_15_)_45_As_30_Cu_25_) is moderate, but its Seebeck coefficient is relatively high. Zhang et al. [48] used it as the functional material and polyethyleneimine (PEI) as the cladding to fabricate the temperature sensor shown in Fig. 2l. The fiber can operate in a wide temperature range of up to 150 °C. A single fiber can quantify the temperature at a fixed position with a temperature resolution as high as 0.05 °C, and it can also locate the heat source at the millimeter level under a fixed temperature. Further, to achieve simultaneous temperature monitoring and localization, a 3 × 3 fiber array was constructed, as shown in Fig. 2m. A spatial temperature resolution of N^2^ can be achieved with only 2N fibers. This fiber array has ultra-high flexibility and can monitor temperature over a large area with high resolution.

In addition to thermistors and the thermoelectric effect, combining capacitors with the interdisciplinary measurement technique of frequency-domain reflectometry [49] can also be used for distributed temperature sensing. A capacitive sensor consists of the upper and lower electrode and a dielectric layer in between. In practical applications, most capacitive sensors can be regarded as parallel-plate capacitors, and the capacitance calculation formula is as follows:

4 $$\mathrm{C} = \frac{{\varepsilon}_{0}{{\varepsilon}}_{\mathrm{r}}{\mathrm{A}}}{\mathrm{d}}$$

In the formula, *C* is the capacitance, *ε*_0_ is the vacuum permittivity, *ε*_r_ is the relative permittivity of the dielectric layer, and *A* and *d* are the facing area and the distance between the upper and lower electrode, respectively. When subjected to external stimuli, *ε*_r_, *A*, and *d* may change, causing a change in capacitance [98]. Capacitive sensors have the advantages of high linearity, low hysteresis, and short response time [99]. However, the capacitance change in microcapacitive sensors is small and easily affected by environmental interference, so accompanying signal processing and amplification circuit devices are required [100].

Time-domain reflectometry (TDR) and frequency–domain reflectometry (FDR) were initially developed to detect fault locations in continuous transmission lines. Both techniques rely on vector network analyzers (VNA). TDR sends a high-frequency pulse along the transmission line. The signal is partially reflected at each point where the impedance changes. The location of the interference can be calculated based on the time when the reflected wave reaches the starting point of the line [101]. Different from TDR, FDR sends a set of wideband sinusoidal swept frequency signals into the line. By comprehensively analyzing and processing parameters such as the standing wave, wideband impedance spectrum, and reflection coefficient spectrum of the reflected wave, the location of the interference can be determined [102]. Capacitance is an important component of impedance changes, and combining capacitors with FDR is an effective way to convert external signals into electrical signal outputs.

The thickness and permittivity of some polymers change with temperature. Yu et al. [49] selected low-density polyethylene (LDPE) with a high thermal expansion coefficient as the sensing material and copper wire as the electrodes to fabricate the fiber shown in Fig. 2n. A thermoelectric cooler (TEC) is used to locally heat the fiber (Fig. 2o). The temperature changes cause alterations in the thickness and permittivity of LDPE, increasing the impedance. Based on the principle of FDR, the reflection signal changes accordingly (Fig. 2p, q), thereby enabling temperature sensing. This method holds great potential for low-cost, distributed temperature sensing.

Table 3 summarizes the performance parameters of temperature sensors based on three different principles. Thermistor-based temperature sensors, with their simple working principle, are the most widely used but exhibit inferior performance. Thermoelectric effect-based sensors achieve the highest resolution and temperature range, surpassing most temperature sensors prepared by other methods [27, 103]. However, their structural complexity limits practical applications. Capacitance temperature sensors enable distributed temperature sensing and simplify system structure, yet their performance requires significant improvement. Future research should focus on enhancing the overall performance of temperature sensors while simplifying their structure and broadening applications.

**Table 3:** Performance parameters of temperature sensors based on three different principles

Principles	Range (°C)	Sensitivity	Resolution (°C)	Response time (s)	References
Thermistor	25–45	− 0.285% °C^−1^	1	11.6	[78]
Thermoelectric effect	25–150	–	0.05	3	[48]
Capacitor	25–85	9.8 × 10^−5^ °C^−1^	2	–	[49]

### Acoustic Sensors

Sound is ubiquitous in nature and human activities. Acoustic sensors play a crucial role in various fields such as natural disaster prediction, ultrasonic imaging of human tissues, and detection of natural gas pipeline leaks [104–106]. The earliest acoustic sensors were rigid [107], which limited their applications. With the development of flexible electronics, the new generation of flexible and wearable acoustic sensors has stood out due to their good flexibility and biocompatibility. The thermal drawing process provides an idea for the large-scale fabrication of acoustic fiber sensors, enhances the applicability of textiles, and can achieve functions such as sound localization and acoustic communication [19, 108].

Most acoustic sensors operate based on the piezoelectric effect. It is a physical phenomenon consisting of two parts: the direct piezoelectric effect and the inverse piezoelectric effect. The direct piezoelectric effect refers to the state where, without external forces, the positive and negative charges within the piezoelectric material are balanced, and the material is electrically neutral. When subjected to external forces, the material deforms, causing the centers of positive and negative charges to shift, resulting in polarization and the generation of positive and negative charges on two opposite surfaces. When the external force is removed, the piezoelectric material returns to its initial state and becomes non-electrified again. The converse piezoelectric effect, on the other hand, means that when a piezoelectric material is placed in an electric field, it undergoes deformation. When the applied electric field is removed, the piezoelectric material returns to its initial state [109].

The principles of the direct and inverse piezoelectric effects are shown by Hooke's law and electrical equations, respectively:

5 $${\mathrm{S}}_{\mathrm{T}} = {\mathrm{d}}_{\mathrm{t}}{\mathrm{E}}$$

6 $${\mathrm{D}}_{\mathrm{E}}= \mathrm{dT}$$

Further linear approximation leads to the first-type piezoelectric equations [110]:

7 $${\mathrm{S}}_{\mathrm{i}} = {\mathrm{s}}_{\mathrm{ij}}^{\mathrm{E}}{{\mathrm{T}}}_{\mathrm{j}}+{\mathrm{d}}_{\mathrm{im}}{{\mathrm{E}}}_{\mathrm{m}}$$

8 $${\mathrm{D}}_{\mathrm{k}} = {\mathrm{d}}_{\mathrm{jk}}{{\mathrm{T}}}_{\mathrm{j}}+{\varepsilon}_{\mathrm{km}}^{\mathrm{T}}{{\mathrm{E}}}_{\mathrm{m}}$$

In the formula, *D* is the electric displacement, *d* is the piezoelectric constant, *T* is the stress, *S* is the strain, *E* is the electric field strength, *s*^*E*^ is the elastic compliance constant under constant electric field strength, *ε*^*T*^ is the permittivity, and *i, j, k* are the coordinate directions. Among these, the piezoelectric constant *d* is the most important parameter for measuring the piezoelectric performance of piezoelectric materials. Sensors based on the piezoelectric effect can be self-powered and feature fast response and high sensitivity [111, 112]. However, due to the unique properties of piezoelectric materials, piezoelectric sensors can only detect dynamic pressure changes and are powerless against static pressure. Currently, piezoelectric polymers represented by polyvinylidene difluoride (PVDF) and poly (vinylidene fluoride-trifluoroethylene) (P(VDF-TrFE)) have been widely used in acoustic sensors. However, their piezoelectric constant is much lower than traditional piezoelectric materials such as piezoelectric ceramics. Therefore, to fabricate higher-sensitivity acoustic sensors, researchers have made many attempts to enhance the piezoelectric performance of fibers by increasing the active area and improving material selection.

**Fig. 3:**
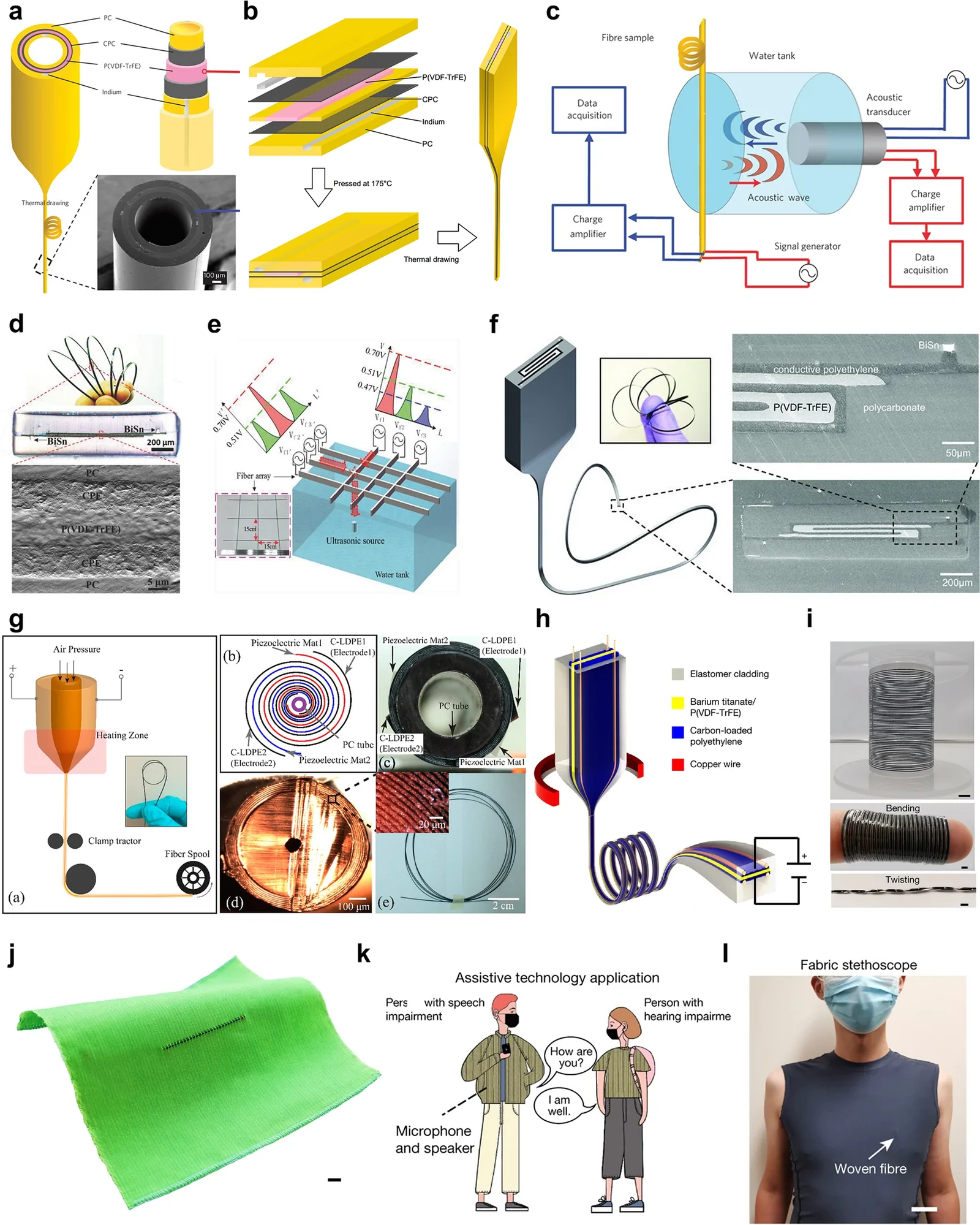
Thermally drawn fibers for acoustic sensing. **a**, **b** Fabrication of cylindrical and rectangular acoustic fibers, both with the same material. **c** Experimental setup for acoustic characterization of acoustic fibers. Reproduced with permission [113] Copyright 2010 Springer. **d**, **e** Fabrication and acoustic sensor performance of piezoelectric fiber that can locate the source of hydroacoustic sound. Reproduced with permission [108] Copyright 2017 Wiley.** f** Fabrication of the acoustic fiber with a folded structure. Reproduced with permission [114] Copyright 2012 Wiley. **g** Fabrication of the acoustic fiber with a "Swiss roll" structure. Reproduced with permission [81] Copyright 2017 American Chemical Society. **h**, **i** Acoustic fiber with BTO/PVDF-TrFE composite as a piezoelectric layer. **j** Acoustic fabric containing a single piezoelectric fiber. **k**, **l** Applications in communication and heart rate detection. Reproduced with permission [19] Copyright 2022 Springer

In 2010, Egusa et al. [113] first fabricated acoustic fiber sensors via thermal drawing process. In this study, the fibers had two structures: rectangular and concentric. Both fibers used the ferroelectric polymer P(VDF-TrFE) as the piezoelectric layer, indium and carbon-loaded poly(carbonate) (CPC) together as electrodes, and PC as the insulating cladding, as shown in Fig. 3a, b. The fibers exhibited piezoelectric response and acoustic transduction over a frequency range from kilohertz to megahertz. Moreover, the PC cladding enabled the fibers to work underwater. As shown in Fig. 3c, the fibers could function as both sensors and actuators, with piezoelectric responses matching the inherent frequency characteristics of the transducers. Furthermore, a Fabry–Pérot interferometer (FPI) structure, which is an optical cavity composed of two parallel mirrors, was constructed in the fibers. Light is reflected and transmitted multiple times inside the interferometer, generating a series of interference fringes. Only specific wavelengths of light are enhanced through interference, while others are suppressed. Thus, the fibers also demonstrated the potential for modulating complex optical devices. Based on the rectangular structure, Wang et al. [108] fabricated an acoustoelectric fiber, as shown in Fig. 3d. It can effectively detect underwater acoustic sources in the frequency range of 2–8 MHz, with a signal-to-noise ratio above 20 dB and the ability to demodulate multiple frequencies simultaneously. Additionally, a two-dimensional fiber array (Fig. 3e) was assembled, capable of locating underwater acoustic sources at the centimeter level.

Thermally drawn fibers have a micrometer-scale cross-sectional area. The small cross-sectional area makes them flexible and easy to assemble, but it also reduces the active area, thereby limiting the piezoelectric performance of the fibers. To increase the active area of acoustic fiber sensors, Chocat et al. [114] designed a folded structure, as shown in Fig. 3f, using P(VDF-TrFE) as the piezoelectric layer and PC as the insulating cladding. To resist capillary breakup while maintaining a certain level of conductivity, the electrodes were composed of low-viscosity carbon-loaded polyethylene (CPE) and BiSn. The uniformity of the fibers was demonstrated through multifiber interference experiments. The extensive contact area effectively enhanced the piezoelectric performance of the fibers, paving the way for acoustic sensors over large areas and even complex three-dimensional structures.

P(VDF-TrFE) can spontaneously crystallize into the β-phase during the drawing and solidification process, avoiding the complex polarization process. However, it is expensive. Additionally, the incorporation of metal electrodes in the studies above limits the flexibility of the fibers. To address these issues while enhancing the piezoelectric performance of the fibers, Lu et al. [81] innovatively fabricated a fiber with a "Swiss roll" structure. They used carbon-impregnated low-density polyethylene (C-LDPE) as the electrode layer and perovskite ceramic (BaTiO_3_ or Pb (Zr_0.52_Ti_0.48_) O_3_) nanoparticles and carbon nanotubes (CNTs) into the PVDF layer, improving the piezoelectric performance while ensuring flexibility. The piezoelectric and electrode layers are alternately arranged to form a multilayer cladding, as shown in Fig. 3g. This structure further increases the piezoelectric material's active area and simplifies the fibers' connection. The square of the fiber's output voltage is proportional to the acoustic wave frequency, making it suitable for sound insulation detection.

The previous research only focused on acoustic fibers without weaving them into fabrics. There are two significant challenges in realizing sensitive acoustic fabrics. Traditional fabrics have the property of damping sound, and acoustic fibers have low sensitivity in the air. Inspired by the eardrum in the human auditory system, Yan et al. [19] innovated from both fiber and fabric levels and realized an acoustic fabric that relies solely on one single piezoelectric fiber. The piezoelectric fiber was fabricated via the thermal drawing process, and its structure is shown in Fig. 3h, i. The piezoelectric layer was composed of barium titanate and P(VDF-TrFE) composite. CPE and four copper wires together served as electrodes, which were asymmetrically encapsulated in the styrene-(ethylene-*co*-butylene)-b-styrene (SEBS) cladding. To enhance the sensitivity of the acoustic fiber, a stepwise polarization method was proposed, where 0 V and gradually increasing voltages were applied alternately. Eventually, the piezoelectric constant *d*_31_ of the fiber reached 46 pC/N. Moreover, a strong coupling between the fiber and the membrane's mechanical vibration mode led to higher electrical output than an individual fiber, further boosting sensitivity. The SEBS ensured the fiber's flexibility, allowing it to be woven into the fabric using a traditional loom. As shown in Fig. 3j, the fabric has the same cotton warp and high-modulus Twaron filament yarns in the weft direction. The fabric exhibited excellent electrical signals within the audible frequency range. The volume of the fiber in the fabric is less than 0.1%. The fiber obtained from a single stretching process can be used to make a fabric covering dozens of square meters. It demonstrated superior performance to acoustic sensors fabricated via other methods [115], while achieving performance levels comparable to commercial microphones. Finally, the acoustic fabric was demonstrated in applications such as sound direction detection, acoustic communication, and heart rate detection (Fig. 3k, l). This scalable acoustic fabric holds promise for advancing the fields of human–computer interaction and physiological monitoring.

### Mechanical Sensors

As a part of flexible electronics, mechanical fiber sensors are not only crucial for detecting various human movements but also key component in flexible robotics. Mechanical sensors mainly include the measurement of three types of deformation: pressure, strain, and bending. Mechanical TDFSs hold great potential in multiple fields, such as Morse code translation, chronic disease detection, smart fabrics, and ion concentration measurement.

**Fig. 4:**
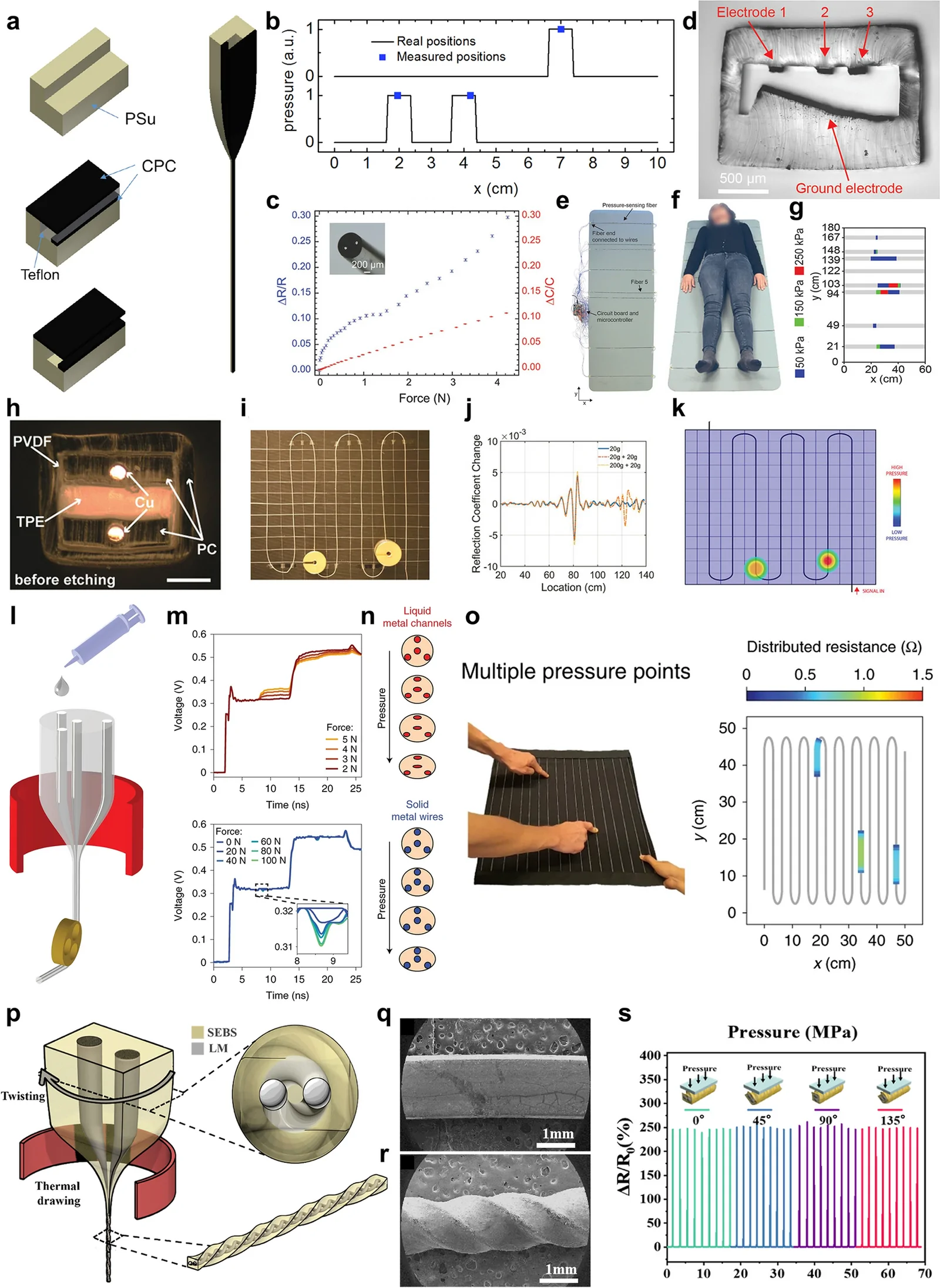
Thermally drawn fibers for pressure sensing. **a** Fabrication of a MEMF. **b** Two-point pressure test. Reproduced with permission [116] Copyright 2017 IOP. **c** TDFS for pressure quantification. Reproduced with permission [72] Copyright 2018 Wiley. **d** Fiber for pressure quantification and location. **e**–**g** Eight pressure fiber sensors are integrated into a gymnastic mat for body posture and motion monitoring. Reproduced with permission [80] Copyright 2020 Wiley. **h** Optical microscopic image of FDR-based fiber cross section. **i**–**k** Physical image, reflection coefficient changes, and reconstruction of pressure map of multipoint pressure testing. Reproduced with permission [49] Copyright 2020 Wiley.** l** Fabrication of a TDR-based fiber. **m** Waveforms of liquid metal top and solid metal bottom triangular lines. **n** Schematic of the cross-sectional deformation mechanism of lines under increasing pressure. **o** Three-point pressure test. Reproduced with permission [118] Copyright 2020 Springer. **p**–**r** Fabrication and morphology of HTLSF. **s** Variation in relative resistance of HTLSF under the same pressure in different directions. Reproduced with permission [79] Copyright 2020 Chinese Society of Metals

The principles of TDFSs for pressure sensing mainly involve resistance and capacitance. The principle of capacitive pressure sensors is analogous to temperature sensors. Unlike temperature sensors, in resistive pressure sensors, the primary contributor to resistance change is the geometric deformation caused by applied pressure. Gorgutsa et al. [61] fabricated a pressure fiber sensor based on capacitance, marking the first attempt of the thermal drawing process in the field of mechanical sensors. The preform was rolled from two dielectric films and two conductive polymer films, forming a "Swiss roll" structure that significantly enhanced capacitance. A single fiber could achieve sliding sensing. However, it could only detect touch points closest to the external electrodes, limiting its applications. To address this issue, Nguyen-Dang et al. [116] developed a pressure fiber sensor with a cantilever structure based on microelectromechanical system (MEMS) (Fig. 4a). Two CPC electrodes with linear resistance were selected. When pressure was applied, two electrodes made partial contact, generating an electrical signal. The signal was linearly correlated with the pressure position at a submillimeter resolution. This fiber could locate two simultaneously applied pressure points, making it the first 1D system capable of locating multiple pressure points without relying on a 2D network (Fig. 4b).

The PSU cladding of microelectromechanical fiber (MEMF) is rigid. With the development of flexible electronics, people are increasingly inclined to choose flexible and stretchable elastomeric materials to expand their applications in wearable devices. Despite more than a decade of research in thermal drawing, selecting an elastomeric cladding that maintains high viscosity during the drawing process remains a challenge. To address this issue, Qu et al. [72, 117] analyzed the rheological properties and microstructures of different materials and developed the rheological criteria of the thermal drawing process. They ultimately demonstrated that various thermoplastic elastomers (TPEs), such as geniomer and SEBS, have the potential to be used as claddings. For example, SEBS fibers can withstand up to 500% elastic deformation. By introducing two parallel liquid metal (LM) electrodes, as shown in Fig. 4c, the pressure was quantified for the first time based on the principles of resistance and capacitance, with a detection limit as low as 0.01N.

Researchers have conducted extensive studies based on TPEs. For example, Leber et al. [80] used SEBS as the cladding and CPE as the polymer electrode. By constructing an asymmetric structure shown in Fig. 4d, they fabricated a fiber sensor capable of simultaneously locating and quantifying pressure. Its signal response showed negligible variation after 10,000 loading–unloading cycles. The fiber, obtained by a single drawing process, was divided into eight strands and integrated into a gymnastics mat (Fig. 4e–g). They can accurately capture the body's position, posture, and movements. Compared to conventional 0D sensor networks, this approach significantly reduced the number of electrical connections. This fiber achieved large-area pressure sensing for the first time, but the pressure resolution was poor. Yu et al. [49] leveraged the high elasticity of TPEs and combined them with the FDR measurement method to preliminarily realize distributed pressure sensing. The fiber featured copper wires as the electrodes and PC as the cladding, with the structure shown in Fig. 4h. When subjected to multipoint pressure, the distance between the parallel copper wires changed, altering the fiber's impedance, which generated multiple reflection signals, thus enabling the precise location of multipoint pressure (Fig. 4i–k). The spatial resolution of the fiber was 2.2 cm, and the pressure resolution was 4 kPa. Leber et al. [118] improved this method by combining the TDR measurement method to fabricate a pressure fiber sensor with liquid metal electrodes arranged coaxially (Fig. 4l). Compared to copper wire electrodes, liquid metal electrodes are more deformable (Fig. 4m, n), enhancing the pressure resolution to 0.2 N. When woven into fabric for testing (Fig. 4o), the fiber could accurately locate and quantify multiple pressure points with a spatial resolution of less than 1 cm. This method further reduced electrical connections, making it highly suitable for flexible wearable devices.

Conventional fiber structures only have sensing capabilities in specific directions. Although previous studies have investigated detecting the direction of pressure [72], isotropic pressure sensors have remained a challenge. Recently, Zhang et al. [79] have fabricated the helical two liquid metal channel SEBS fibers (HTLSFs) (Fig. 4p–r). The unique structure provides the fibers with over 1000% strain and excellent electrical conductivity, allowing them to sensitively respond to both small and dynamic pressures over a wide range. Furthermore, when pressure is applied in different directions, the change in fiber resistance is almost identical (Fig. 4s), extending the application scenarios of pressure TDFSs in complex environments.

**Fig. 5:**
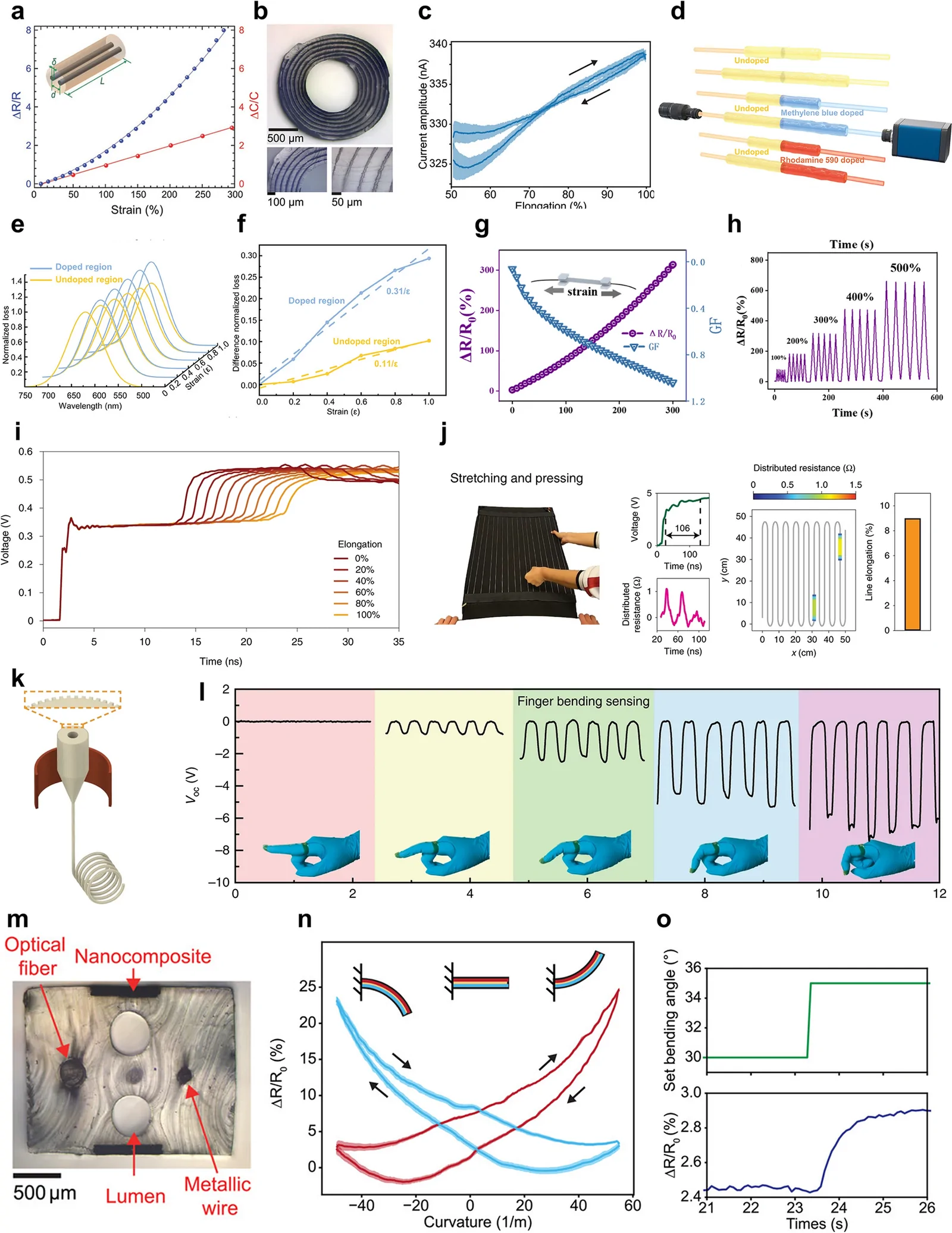
Thermally drawn fibers for strain and bending sensing. **a** Structure and performance of resistive and capacitive strain sensors. Reproduced with permission [72] Copyright 2018, Wiley. **b, c** Optical image and strain sensing performance of a capacitive strain fiber sensor. Reproduced with permission [73] Copyright 2023 Wiley.** d** Schematic of the testing method for photoelectric strain sensors. **e, f** Normalized absorption spectra variation of the strain sensor and the difference normalized loss. Reproduced with permission [82] Copyright 2024 Wiley. **g** Variation in relative resistance and GF value of HTLSF with respect to tensile strain.** h** Relative resistance changes of HTLSF at different strains. Reproduced with permission [79] Copyright 2020 Chinese Society of Metals. **i** Waveform changes with elongation. **j** Simultaneous measurement of multipoint pressure and strain. Reproduced with permission [118] Copyright 2020 Springer. **k** Fabrication of a TENG fiber with micron-scale surface textures. **l** Finger bending sensors. Reproduced with permission [127] Copyright 2020 Springer. **m** Optical image of a fiber cross section, integrating two nanocomposite electrodes. **n** Relative resistance changes versus the fiber curvature for both nanocomposite electrodes. **o** Relative resistance changes of the nanocomposite to a small bending angle step of 5°. Reproduced with permission [73] Copyright 2023 Wiley

The principles of TDFSs for strain sensing mainly involve resistance, capacitance, and photoelectricity. Only after the thermal drawing rheological criteria were proposed that researchers began studying strain sensors. By incorporating two parallel liquid metal electrodes into SEBS, a strain sensor was easily fabricated based on resistance and capacitance principles (Fig. 5a) [72]. Capacitive strain sensors have good linearity but small changes in capacitance. To improve the signal response amplitude, Leber et al. [73] used nanocomposites as electrodes to fabricate a fiber with a structure resembling an axial lead capacitor (Fig. 5b). This special structure increased the fiber’s capacitance by 13 times compared to the standard parallel-plate structure. Figure 5c shows the current variation of the fiber with an elongation rate between 50 and 100%, demonstrating good strain sensing performance.

In addition, photoelectricity is also applied in strain sensors. By utilizing the difference in refractive indices of two types of SEBS, light loss under different deformations can be measured to detect strain changes [119]. As a type of TPE material, polyurethane (TPU) has self-healing properties after special treatment. Based on this, Qi et al. [82] proposed a structurally simple self-healing fiber. An original STPU fiber was spliced with a methylene blue-doped STPU fiber and tested in the device shown in Fig. 5d. As strain increased, the normalized loss of both fibers increased, with the methylene blue-doped fiber showing more significant changes (Fig. 5e, f). The fiber exhibited a nearly linear response across the 100% strain range, making it suitable for distributed strain sensing. Moreover, with the self-healing feature, the fiber is more durable, making it convenient for long-term application. The HTLSF introduced in the pressure sensing section can also be used as a strain sensor [79]. Compared to previous studies, its strain range has been increased to 500% (Fig. 5g, h). However, it cannot simultaneously measure strain and pressure. In the TDR-based fiber, as the elongation increases, the signal transmission time also increases, enabling strain sensing with a strain resolution of 0.25% (Fig. 5i). Additionally, by decoupling the reflection signals, simultaneous measurement of multipoint pressure and strain can be achieved [118] (Fig. 5j). This represents a groundbreaking breakthrough in mechanical sensing, providing a feasible solution for the application of multifunctional TDFSs in flexible wearable devices and robotics.

Table 4 summarizes the performance parameters of pressure and strain sensors based on different principles. Resistive pressure sensors exhibit a lower theoretical spatial resolution, but they cannot simultaneously detect more than two pressure points. Capacitive pressure sensors possess distributed sensing capabilities, yet they have a narrower measurement range. Capacitive and optical strain sensors demonstrate comparable performance but differ in applications. Capacitive strain sensors prove more cost-effective for large-scale distributed sensing, while optical strain sensing is better suited for non-contact measurement. In summary, thermally drawn pressure sensors show superior comprehensive performance compared to those fabricated by other methods [120]. However, the performance of strain sensors still require further improvement.

**Table 4:** Performance parameters of pressure sensors and strain sensors

Sensor types	Principles	Range	Sensitivity	Resolution	Spatial resolution	References
Pressure sensors	Resistance	–	0.3 N	–	0.75 mm	[116]
	Capacitance	> 5 N	20 mV N^−1^	0.2 N	< 1 cm	[118]
Strain sensors	Capacitance	100%	0.1 ns %^−1^	0.25%	–	[118]
	Photoelectricity	100%	0.31/ϵ	–	–	[82]

The principles of TDFSs for bending sensing mainly involve resistance and triboelectric nanogenerators (TENGs). TENGs convert mechanical energy into electrical energy through by coupling contact electrification and electrostatic induction [121]. When external stimuli are applied to a TENG, materials with different electron affinities contact, generating opposite charges. After the external force disappears, the materials separate, and electrons in the external circuit flow to balance the potential, generating an electrical signal. When external force is reapplied, electrons flow in the opposite direction. When the two materials come into contact again, the induced charges are neutralized, and there is no electron flow [122]. The voltage output formula of TENGs is as follows [123]:

9 $$\mathrm{V}=-\frac{1}{{\mathrm{C}}\left({\mathrm{X}}\right)}\mathrm{Q} + {\mathrm{V}}_{\mathrm{oc}}\left({\mathrm{X}}\right)$$

In the formula, *V* is the total voltage, *C* is the capacitance, *X* is the distance between two electrodes, *Q* is the charge transferred between the electrodes, and *V*_oc_ is the contribution to the voltage from the polarized triboelectric charge. TENG has a wide range of material choices, a simple structure, low cost, and the ability to self-power, making them an ideal alternative to piezoelectric sensors [91, 124]. However, the sensing performance of TENGs is easily influenced by environmental factors, and they are only sensitive to dynamic pressure. Theoretically, materials with a greater difference in electron affinity generate a larger voltage [125, 126]. Additionally, altering the surface roughness of materials is another method to enhance voltage output.

Dong et al. [127] found that geniomer, as a TPE, also possesses negative triboelectric polarity, making it suitable cladding for TENG fibers. Based on this, they integrated liquid metal electrodes and fabricated a TENG fiber with micron-scale surface textures (Fig. 5k). The fiber exhibited enhanced open-circuit voltage with operational stability over 40,000 cycles. When attached to a glove, the fiber's voltage output varied with the bending angle (Fig. 5l). The above research only roughly described how the fiber's voltage output changes with the bending angle, without quantification. Leber et al. [73] used nanocomposites as electrodes to fabricate a fiber with the structure shown in Fig. 5m. At different bending angles, the upper and lower electrodes are in stretching and compressing states, respectively, causing changes in resistance and enabling quantification of the bending angle (Fig. 5n). As shown in Fig. 5o, the fiber can detect bending changes as small as 5°. Overall, the development of bending sensors is still in its infancy. Their sensitivity, response time, and other performance aspects need to be improved.

### Chemical Sensors

Based on specific sensing materials, chemical sensors enable qualitative and highly sensitive quantitative detection of target analytes, with broad applications spanning biomedical diagnostics, environmental monitoring, food safety, and hazardous substance detection [50, 83, 128, 129]. Thermal drawing process can form specific micro–nanostructures, providing a novel fabrication process for highly sensitive, low-cost miniaturized chemical sensors. Based on different signal transduction mechanisms, the chemical TDFSs developed so far can be divided into two categories: photochemical sensors and electrochemical sensors, each with unique detection capabilities and application potential.

**Fig. 6:**
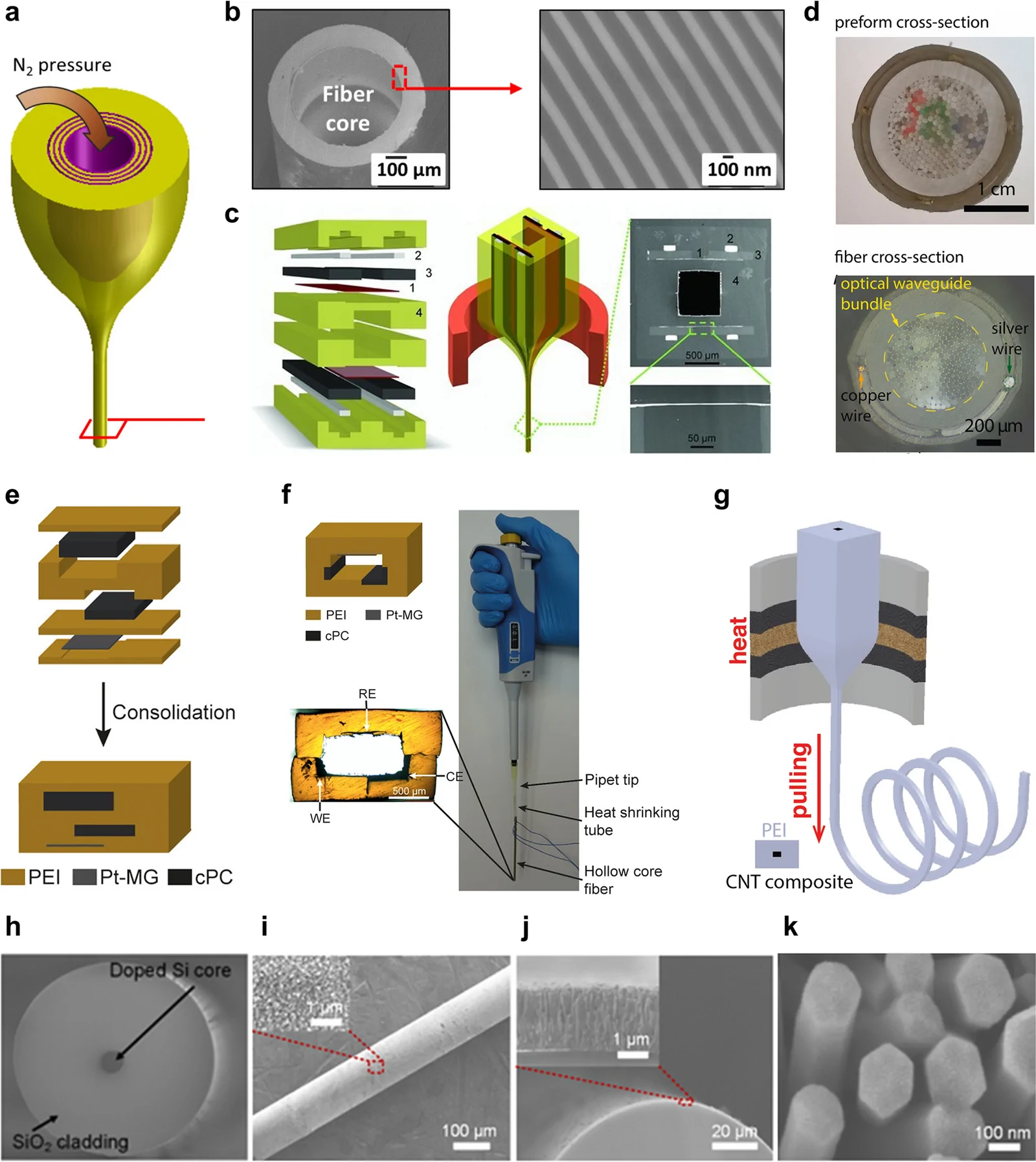
Thermally drawn fibers for chemical sensing. **a**, **b** Fabrication and principle of the PBG fiber. Reproduced with permission [131] Copyright 2012 The Optical Society. **c** A completely distributed chemical sensor. Reproduced with permission [50] Copyright 2012 Wiley. **d** PH probe based on LAPS. Reproduced with permission [133] Copyright 2021 Elsevier. **e** Preform-making process of a three-electrode electrochemical sensor. **f** Cross section of the hollow core fiber. Reproduced with permission [83] Copyright 2021 American Chemical Society.** g** Fabrication of the CNT composite fiber. Reproduced with permission [134] Copyright 2019 Multidisciplinary Digital Publishing Institute. **h**–**k** SEM images of the Co-doped ZnO NRs chemical sensors. Reproduced with permission [135] Copyright 2023 American Association for the Advancement of Science

Light is an effective form of noninvasive energy for sensing various stimuli. Currently, photochemical sensors mainly achieve sensing functions by detecting changes in light wavelength (∆*λ*). Photonic bandgap (PBG) fibers control the propagation of light by designing periodic structures with different refractive indices. Only light outside the bandgap within a specific wavelength range can be reflected [130]. Stolyarov et al. [131] combined the principles of chemiluminescent (CL) reactions and PBG fibers, fabricating a hollow multilayer PBG chemical fiber sensor (Fig. 6a, b). During the drawing process, external gas was introduced into the hollow fiber under controlled pressure to modulate the optical transmission bandgap, increase the core diameter, and reduce transmission loss. Meanwhile, the CL material was centrally placed inside the fiber, enhancing detection sensitivity. The CL material reacted exclusively with hydrogen peroxide (H_2_O_2_) vapor, which avoided the influence of environmental factors on the experimental results. The minimum detectable concentration of H_2_O_2_ vapor by the sensor reached 100 ppb. However, limitations such as the fiber’s numerical aperture, transmission, and bending loss restrict the detection distance and sensitivity of photochemical sensor. To overcome these drawbacks, Gumennik et al. [50] adopted a monolithic integration approach using Se_97_S_3_ as the CL material to achieve distributed chemical sensing along the entire fiber length. The preform (Fig. 6c) integrated two independent optical detection units distributed on either side of a rectangular hollow core, further improving detection sensitivity. Se_97_S_3_ also reacted exclusively with H_2_O_2_, and its minimum detectable concentration for hydrogen peroxide is reduced to 10 ppb. This fiber sensor provides a concept for long-range and distributed sensing over hundreds of meters, with sensing capabilities comparable to films [132]. Overall, photochemical sensors exhibit high sensitivity, low signal transmission loss, and extremely low detection limits.

Electrochemical sensors are widely recognized for their simplicity in testing and low cost, making them particularly advantageous in quantitative analysis. Measuring pH in vivo has long been a challenge. Guo et al. [133] developed an integrated pH probe for spatial pH sensing (as shown in Fig. 6d) based on a light-addressable potentiometric sensor (LAPS). The Si_3_N_4_ sensing layer specifically responds only to H⁺ ions, showing negligible interference from other ions (such as Na⁺, K⁺). The fiber can simultaneously measure pH variations across 14 pixels without environmental interference, achieving a spatial resolution of 250 μm and a temporal resolution of 30 Hz. This novel label-free chemical sensor is significant for revealing the relationship between brain neural activity and pH changes.

Richard et al. [83] fabricated a three-electrode electrochemical fiber sensor. In the preform shown in Fig. 6e, Pt_57.5_Cu_14.7_Ni_5.3_P_22.5_ (Pt-MG) was used as a pseudo-reference electrode, and two carbon-black-filled conductive polycarbonate (cPC) films were used as working and counter electrodes. The cPC surfaces were chemically treated to enhance electrochemical activity. To further improve sensitivity, a capillary structure with a high surface area was fabricated (Fig. 6f). Using cyclic voltammetry (CV) or chronoamperometry (CA) for testing, the fiber exhibited sensitivity comparable to commercial screen-printed electrodes (SPE), demonstrating its ability to detect acetaminophen. Furthermore, after functionalizing chemical sensors with different ion recognition elements, they can also quantify ion concentrations. For instance, inspired by nanocomposites, Nishimoto et al. [134] incorporated (CNTs) into carbon-black-impregnated polyethylene (CB-CPE) to fabricate a composite fiber (Fig. 6g). The high catalytic activity of CNTs enables them to preferentially catalyze the oxidation of dopamine (DA) at a fixed potential (0.6 V), while other interfering species exhibit low oxidation currents under this potential. By adjusting the CNTs content, they significantly improved the fiber's viscoelastic compatibility and electrochemical sensing performance. In vitro, the detection limit for DA reached as low as 10 nM. After functionalizing the fiber with ion-sensitive membranes (ISM), it could selectively monitor Na^+^ concentrations. Additionally, by combining the principles of metal oxide semiconductor (MOS) sensors, gas detection is feasible. Niu et al. [135] in situ grew Co-doped ZnO nanorods on the surface of Si/SiO_2_-doped fibers (Fig. 6h–k). Co doping reduced the operating temperature of ZnO and preferentially enhanced its selectivity toward CH_4_. At 50 °C, the fiber achieved a maximum detection sensitivity of 16% for 1000 ppm CH_4_. Integrated into miners' clothing, the sensor can monitor CH_4_ concentrations in real time, ensuring safety in mines. Furthermore, Iwama et al. [58] combined a closed bipolar electrode (cBPE) array with the thermal drawing process to develop an electrochemical imaging system capable of 2D imaging of chemical signals within organisms. Overall, electrochemical sensor technology is relatively mature, offering the advantages of low cost and good stability.

### Biosensors

Flexible biosensors are a crucial component in modern biomedical fields [136, 137]. Multifunctional thermally drawn fibers, with excellent flexibility, biocompatibility, and controllable dimensions, can minimize foreign body reactions, making them highly suitable for implantable and wearable biomedical devices [138, 139]. Among these, implantable neural probes, as key tools for studying neurological disorders, have garnered significant attention [140]. However, the elastic modulus of commonly used silicon-based materials is much higher than that of neural tissue. Over prolonged use, this discrepancy can lead to a decline in the signal-to-noise ratio (SNR) and even neuronal damage, ultimately compromising the device’s longevity [141–143] and hindering advancements in the field of flexible neural probes. Consequently, developing neural probes with high SNR, biocompatibility, and long-term stability holds profound significance.

With the development of fluorescence sensors and optogenetics [144, 145], using light-sensitive proteins (e.g., Channelrhodopsin-2), probes have enabled precise stimulation or inhibition of specific neuronal activities. Light has thus become the primary method for neural probe stimulation. To enhance biocompatibility and extend the life span of probes, two main solutions have emerged.

**Fig. 7:**
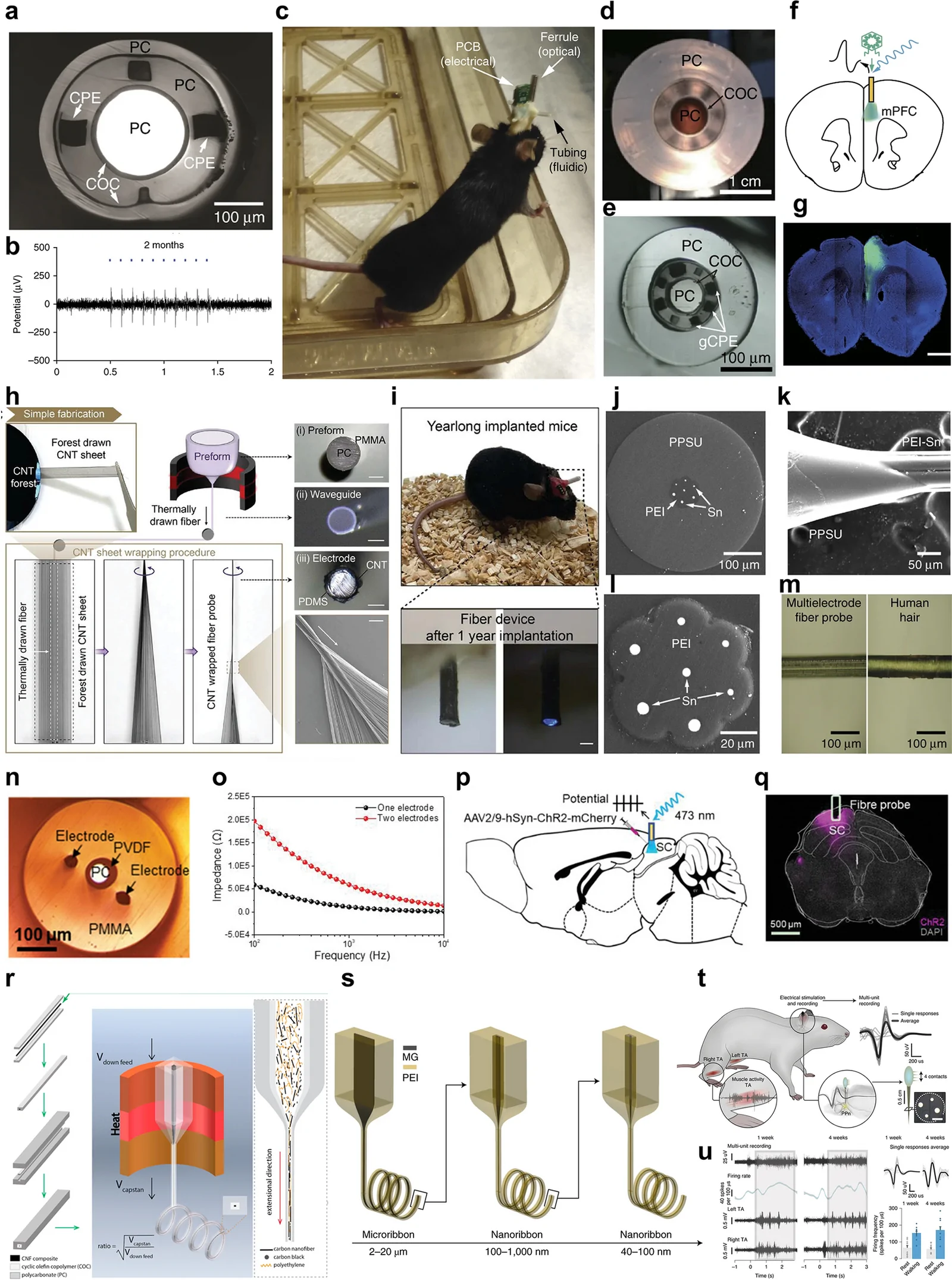
Thermally drawn fibers for biosensors. **a** The cross-sectional image of the multimodality fiber. **b** Electrophysiological recording status after two months. **c** Multimodality fibers were implanted into mice. Reproduced with permission [71] Copyright 2015 Springer. **d**, **e** Cross-sectional images of the preform and the multimodal fiber with gCPE electrodes. **f**, **g** Schematic of viral delivery via the probe. Reproduced with permission [147] Copyright 2017 Springer. **h** Fabrication of the SAMP. **i** The image of the SAMP implanted mice for one year. Reproduced with permission [84] Copyright 2024 Wiley. **j** The SEM cross-sectional image of a multielectrode probe. **k**–**m** After etching PPSU, the multielectrode probe has a diameter of approximately 85 μm. Reproduced with permission [71] Copyright 2015 Springer. **n** The Image of a fiber with two metal electrodes.** o** Impedance of different electrodes in fiber. **p**, **q** The image of the probe implanted into the SC. Reproduced with permission [153] Copyright 2020 Wiley. **r** Fabrication of the composite fiber. Reproduced with permission [85] Copyright 2021 American Chemical Society. **s** Fabrication of a nanoscale ribbon via multistep drawing. **t**, **u** Applications of electrical stimulation and recording based on MG fibers. Reproduced with permission [51] Copyright 2022 Springer

One solution is to fabricate probes entirely from flexible materials [146]. In 2015, Canales et al. [71] fabricated a neural probe that combined PC and conductive polyethylene (COC) to provide optical waveguides, with CPE as the polymer electrode. The probe also incorporated microfluidic channels, as illustrated in Fig. 7a. It can perform optogenetic stimulation, electrical neural recording, and drug delivery simultaneously. Tests on an adult mouse showed that the probe could operate stably for up to two months (Fig. 7b, c). In neural probes, extracellular electrophysiological recordings typically require electrodes with an impedance of less than 2 MΩ [139]. However, CPE electrodes exhibit poor conductivity. When meeting the impedance requirement, the electrode dimensions tend to be larger, which may induce foreign body reactions. Therefore, it is necessary to find highly conductive flexible electrodes to replace CPE. Park et al. [147] used a similar structure (Fig. 7d, e) and doped 5% graphite into the CPE (gCPE) to reduce impedance. The resulting neural probe had a diameter of less than 200 µm and a life span of three months. Figure 7f, g show the probe's functionality for viral delivery. Lu et al. [148] coated conductive silver nanowires (AgNWs) around the coaxial probe to enhance the conductivity of the probe. Although AgNWs don't belong to polymers, their incorporation maintains the probe's excellent flexibility. In tests in freely moving mice, the fibers could stimulate certain muscle groups, making them useful for research on neural pathways after spinal cord injury. CNTs, known for their excellent conductivity, biocompatibility, and electrochemical properties, have been widely studied since their discovery [149–151]. Jeon et al. [84] initially thermally drew PC and PMMA coaxial fiber to provide optical waveguides and then wrapped the forest-drawn CNT sheets around the fibers (Fig. 7h). The neatly aligned CNT sheets ensured excellent electrical signal recording. This structurally aligned multifunctional neural probe (SAMP) solved the problem of co-drawing high-conductivity electrodes with polymer. Moreover, the probes maintained stable function for up to one year (Fig. 7i), marking an unprecedented breakthrough in thermally drawn neural probes and surpassing most implantable probes [152].

Metal electrodes exhibit excellent conductivity but suffer from a large elastic modulus, poor flexibility, and low biocompatibility. Additionally, they typically have a high *T*_m_, making it difficult to integrate with a polymer with low light transmission loss [138]. Therefore, a second solution has emerged, focusing on low *T*_m_ metal electrodes while minimizing electrode dimensions.

Canales et al. [71] used PEI as the cladding and fabricated polymer–metal multielectrode fiber probes through a two-step thermal drawing process. The diameter of the Sn electrode was reduced to 5 µm (Fig. 7j). After selectively etching polyphenylene sulfone resins (PPSU), the fiber size was reduced to 85 µm (Fig. 7k–m). Compared to conventional microwire electrodes, the smaller multielectrode probe induced fewer foreign body reactions, exhibited better biocompatibility, and had a life span of up to three months. Du et al. [153] designed a double-clad waveguide structure using PVDF and PMMA to improve the probe's optical transmission performance (Fig. 7n). Two metal wire electrodes significantly reduced the impedance (Fig. 7o). Figure 7p, q shows a schematic of neural probe implantation into the superior colliculus (SC). The unique material and design enabled long-term (at least 10 weeks) simultaneous optical stimulation and neural signal recording with a high signal-to-noise ratio (SNR = 30 dB). Additionally, low-melting-point indium (In) can also be co-drawn with polymer [154]. A probe incorporating three In electrodes has been demonstrated to be compatible with magnetic resonance imaging (MRI), enabling real-time visualization of brain fluid dynamics. Table 5 summarizes representative works of the two strategies from the perspectives of material selection, size, and service life.

**Table 5:** Summary of the thermally drawn optogenetic stimulation neural probes

Optical waveguides		Electrodes		Size	Service life	References
Material	Loss	Material	Impedance			
*Polymer electrodes*						
PC/COC	2.7 dB cm^−1^	CPE	0.5–2.5 MΩ	d = 400 μm	2 months	[71]
PC/COC	1.5 dB cm^−1^	gCPE	0.62 ± 0.23 MΩ	d = 180–220 μm	3 months	[147]
PC/PMMA	0.769 dB cm^−1^	CNT sheet	0.22 MΩ	d = 160 μm	1 year	[84]
*Metal Electrodes*						
–	–	Sn	0.9 MΩ	d = 85 μm	3 months	[71]
PC/PVDF	1.18 dB cm^−1^	Pt/Au/Cu	22.71 MΩ cm^−2^	d = 200 μm	10 weeks	[153]

Neural probes can also control neuronal activity through electrical signals [155]. To improve biocompatibility and life span, electrical stimulation neural probes also need to enhance flexibility and reduce size. By doping CPE with 2% CNFs, which align in situ during thermal drawing (Fig. 7r), the conductivity of the electrode is further improved. The size of the neural probe is reduced to less than 100 µm × 100 µm, and the recording sites are comparable in size to individual neurons [85]. This microelectrode fiber can record high-quality neural activity from neurons near the electrode during long-term implantation, making them suitable for chronic electrophysiological studies and neurorehabilitation applications.

Metallic glass (MG), as an amorphous metal material, can better balance conductivity and size, along with high chemical stability and oxidation resistance, making it particularly advantageous for neural probes. Yan et al. [51] achieved the controlled fabrication of uniformly ordered MG fibers through multiple thermal drawing steps, reducing the size to approximately 40 nm (Fig. 7s). They further validated the feasibility of this size using principles of fluid dynamics. On this basis, they created probes with four MG electrodes embedded in PEI, enabling stable electrical stimulation and neural activity recording (Fig. 7t, u). This material provides a new approach to enhancing the life span of multimodal neural probes. Additionally, probes fabricated by combining the thermal drawing process and femtosecond laser micromachining technology can achieve electroporation, showing potential for deep brain applications [156].

Overall, improving the biocompatibility of implantable neural probes is a key research focus in the field of biosensors, and the most crucial metric for measuring biocompatibility is the service life. Utilizing polymer materials and reducing electrode size while meeting impedance requirements is an effective way to minimize foreign body reactions and extend the service life. Currently, a service life of up to one year can already meet the needs of many applications [84]. However, for use in the human body, it is necessary to search for materials with even higher biocompatibility to further increase the upper limit of the service life.

In addition to implantable neural probes, thermally drawn fiber biosensors can also detect cell concentrations. Kanik et al. [86], combining microfluidic technology and TENG, fabricated a hollow PVDF fiber. By utilizing liquid for continuous medium exchange, the fiber not only achieved self-powering but also enabled biochemical analysis, allowing for the quantitative detection of ethanol and Escherichia coli cell concentrations. In the future, this innovation holds promise for expanding the range of detectable cells and advancing research in self-powered biosensors.

### Optoelectronic Sensors

Optoelectronic sensors convert optical signals into electrical signals through photosensitive elements and can be used in fields such as ambient light detection and optical imaging. Semiconductors serve as the core material in these devices [25, 157, 158]. Among various semiconductors, glass semiconductors with low drawing temperatures and controllable fluid behavior are more compatible with common cladding and thus widely used in thermally drawn fibers [28, 159, 160]. However, their inferior electrical performance and damage resistance have limited the development of thermally drawn optoelectronic sensors, which will not be discussed here. This section will only introduce two fibers that can effectively improve the performance of thermally drawn optoelectronic sensors.

**Fig. 8:**
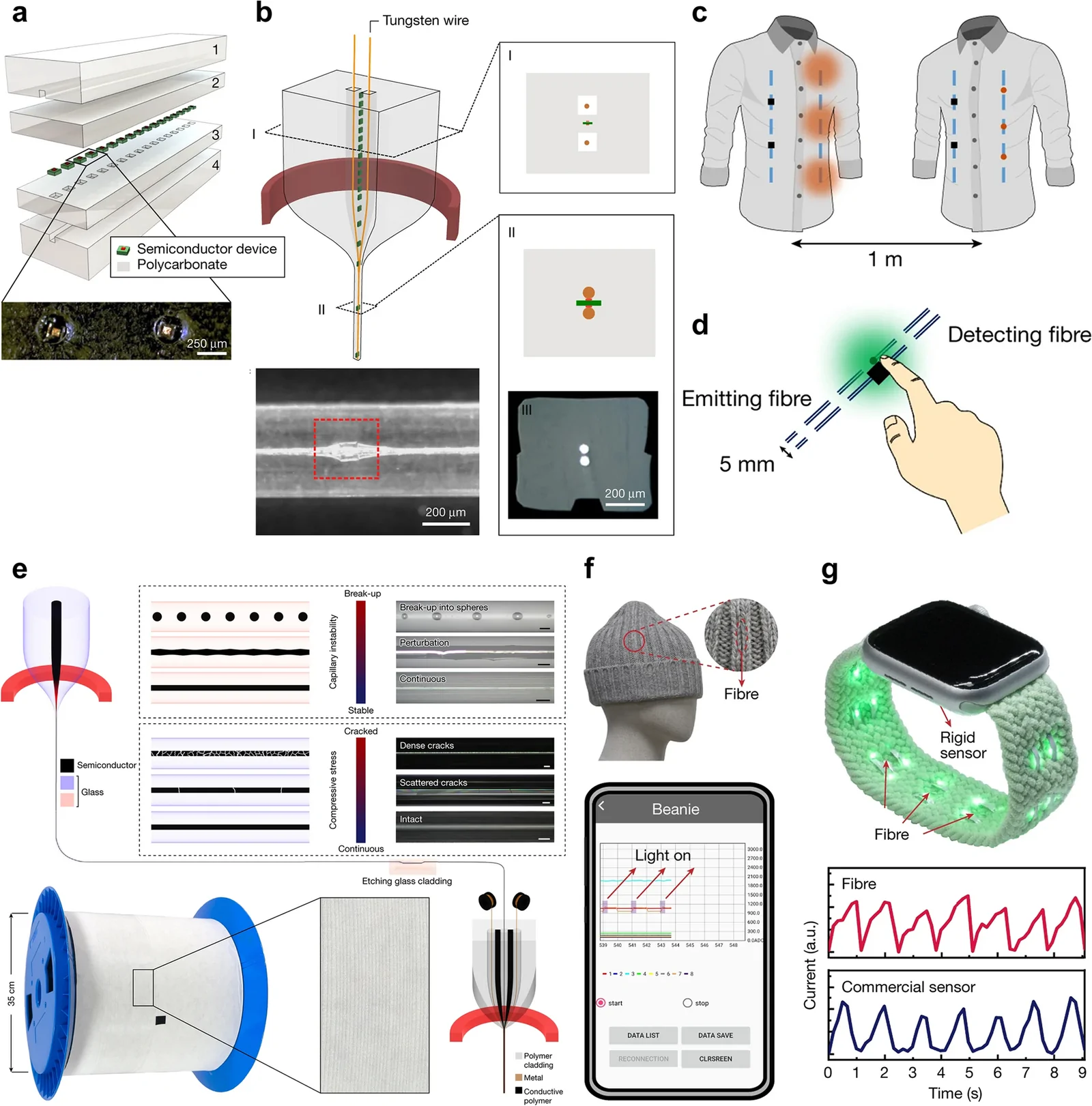
Thermally drawn fibers for Optoelectronic sensing. **a**, **b** Fabrication of the diode fiber. **c** Schematic of the optical communication. **d** Application for heart rate detection. Reproduced with permission [43] Copyright 2018 Springer. **e** Fabrication of the semiconductor optoelectronic fiber. **f**, **g** Optoelectronic fabrics for ambient light detection and heart rate monitoring. Reproduced with permission [87] Copyright 2024 Springer

In 2018, Rein et al. [43] left grooves at fixed intervals in the preform to place semiconducting diodes (Fig. 8a). During the drawing process, the diodes separated axially, and the metal wire electrodes contracted transversely until they established electrical contact with the diodes (Fig. 8b). This method only altered their relative positions without changing their dimensions. By integrating light-emitting and photodetecting p–i–n diodes using this method, light-emitting fiber and photodetecting fiber were fabricated. The fiber exhibited optical detection abilities similar to the internal diodes. Its performance was enhanced by several orders of magnitude compared to photodetectors based on chalcogenide semiconductors. Furthermore, the fiber can be woven into fabrics and maintain stability even after several machine washes. By weaving light-emitting fiber and photodetecting fiber into two fabrics spaced a meter apart, optical communication functionality was achieved (Fig. 8c). Additionally, the fibers can detect heart rate (Fig. 8d). This pioneering method further improves the device integration and functionality diversity in thermally drawn fibers, demonstrating the development potential of the "Moore's law for fibers."

In addition to directly integrating diodes, analyzing the thermal drawing criteria of crystalline semiconductors such as Si and Ge and finding suitable materials and methods are also significant for improving the performance of thermally drawn optoelectronic sensors. Recently, Wang et al. [87] have deeply analyzed the potential issues during the thermal drawing of Si and Ge, including cracks and fractures caused by residual stress, capillary instability phenomena, and so on (Fig. 8e). They attributed the causes to the mismatch of the thermal expansion coefficients between the glass cladding and the semiconductor core. To achieve a stable drawing of Ge semiconductor fibers, they replaced the SiO_2_ cladding with aluminosilicate glass (ASG), which has an annealing point and a thermal expansion coefficient closer to Ge. With this material improvement, ultra-long, continuous, and fracture-free semiconductor fibers were achieved in a single thermal drawing step. The semiconductor fibers with removed cladding were further drawn with metal wires, conductive polymers, and insulating polymers into optoelectronic fibers with single-core and double-core structures. The fibers exhibited performance comparable to commercial planar optoelectronic sensors and excellent flexibility. After being woven into fabrics, they can be applied in various fields, such as ambient light detection and heart rate monitoring (Fig. 8f, g). This study provides a fundamental explanation of the fluid dynamics principles behind thermally drawn semiconductor fibers and advances the development of semiconductor materials in flexible sensors.

### Other Emerging TDFSs

Thermal drawing process is an emerging large-scale fiber fabrication process, and many types of TDFSs are still in their early development. This section provides a brief overview of TDFSs for flow, magnetic, and humidity sensing, which have relatively less research, offering some guidance for researchers who wish to work in these fields.

**Fig. 9:**
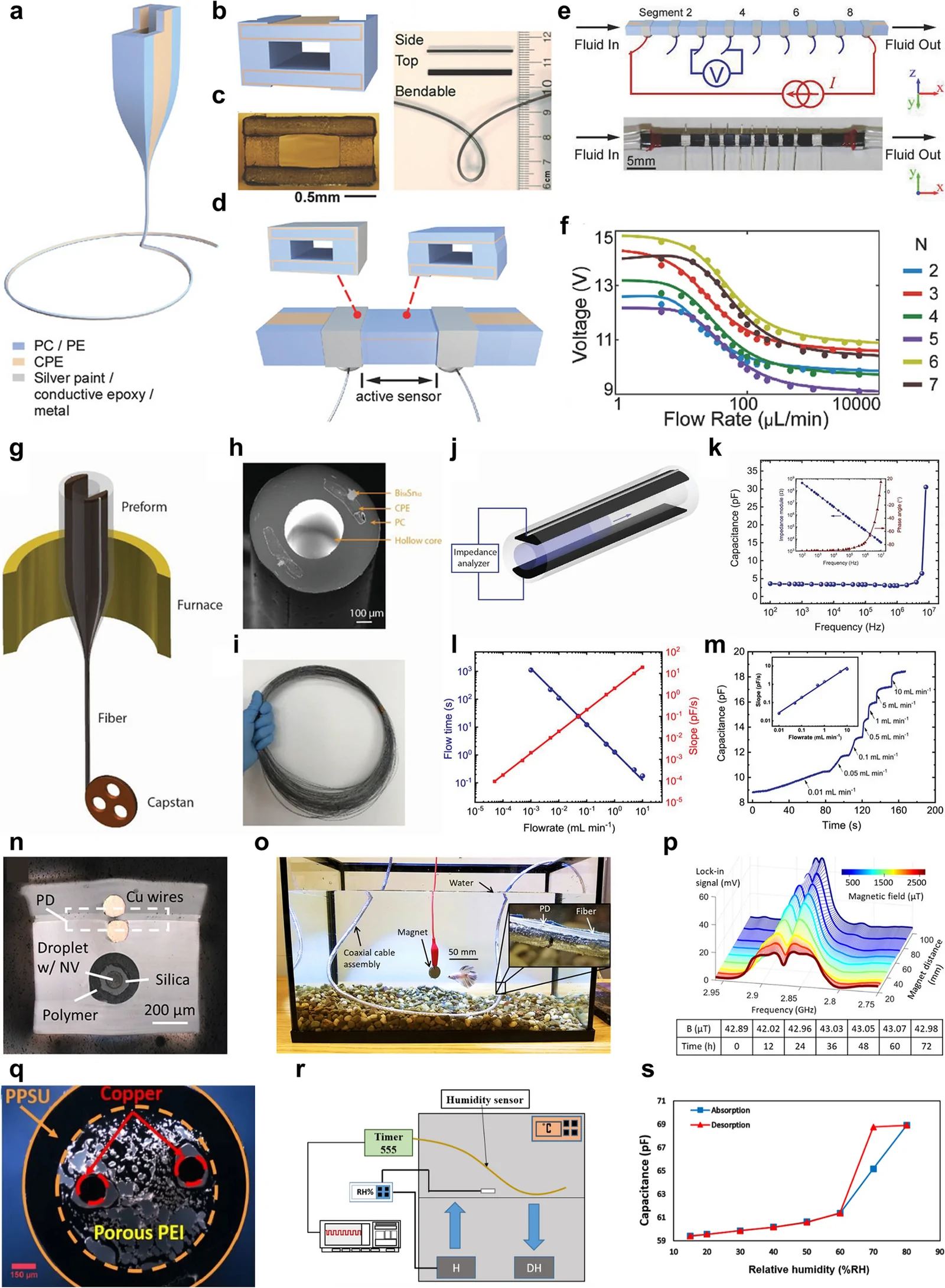
Thermally drawn fibers for flow, magnetic and humidity sensing. **a**–**d** Fabrication of a flow fiber sensor. **e**, **f** Schematic of eight-segment fiber sensor flow test. Reproduced with permission [167] Copyright 2018 Wiley. **g**–**i** Fabrication of a capacitive flow sensor. **j**, **k** Capacitive performance of the fiber. **l**, **m** Flowrate detection of the fiber. Reproduced with permission [52] Copyright 2019 Wiley. **n** The cross-sectional image of the magnetic sensor. **o**, **p** Underwater magnetometry testing. Reproduced with permission [171] Copyright 2019 Wiley. **q** The cross-sectional image of the humidity sensor. **r** Schematic of the humidity measurement device. **s** Capacitance changes under different RH. Reproduced with permission [53] Copyright 2019 Multidisciplinary Digital Publishing Institute

Flow sensors, capable of locating fluid positions and analyzing flow rates, are critical components in clinical medicine, drug delivery, and environmental monitoring [161–163]. Their sensing principles include thermal flow, capacitance, and TENG [164–166]. Among various principles, thermal flow sensor offers advantages in sensitivity, measurement range, and power consumption. However, its sensitivity, pressure drop, temperature rise, and measurement range influence each other, making it challenging to improve overall performance. To address this, Chen et al. [167] drew CPE, a conductive polymer with both high resistivity and large TCR, into hot films (Fig. 9a-d). The resulting fiber sensor maintained a flow velocity sensitivity of 384 mV min µL⁻^1^ while keeping the maximum temperature rise to only 20 °C, which is 5–10 times lower than typical MEMS sensors. Additionally, a multisegment structure was designed to enable high-resolution flow velocity detection within the range of 5–200 µL min⁻^1^ (Fig. 9e, f). Although thermal flow sensors have a slight advantage in measuring varying flow velocities, they have some drawbacks. For instance, to avoid impurities affecting measurement accuracy, the fluid medium is ideally a pure liquid, and inevitable thermal losses can also affect the measurement results, limiting the detection of low flow rates. To overcome these limitations, Dong et al. [52] developed a microflow sensor based on capacitance. The fiber is hollow, with two CPE electrodes embedded in the PC cladding to provide capacitive signals (Fig. 9g–k). The sensor not only detected the position of the front fluid but also expanded the flow velocity range to 50 nL min⁻^1^–10 mL min⁻^1^ (Fig. 9l, m) and can even estimate the liquid concentration. Such a simple and scalable fiber sensor is crucial for detecting fluid signals in biomedical applications.

Magnetic sensors can detect magnetic field strength and direction, enabling the localization of object positions [168]. Due to the spatial distribution characteristics of magnetic fields, magnetic sensors are widely used in non-contact detection platforms [169]. In magnetic sensors, magnetic electronic components play a crucial role. Nitrogen vacancy (NV) quantum magnetometers, as one of these components, demonstrate unique advantages in magnetic sensing due to their spin-based quantum properties [170]. However, integrating optical, microwave, and magnetic excitation in a single system presented challenges, which limited the application of NV-based distributed sensing. Maayani et al. [171] addressed this issue through a two-step thermal drawing process. First, they fabricated a hollow silicon fiber by high-temperature drawing, allowing the incorporation of NV-containing diamond liquid. Afterward, a polymer coating was applied to protect the fiber. This fiber was then drawn along with multiple photodiodes (PDs), copper wires, and PC cladding (Fig. 9n). Finally, the composite fiber was embedded in a larger coaxial cable for microwave (MW) excitation. The resulting distributed magnetic sensor achieved high-sensitivity magnetic measurements over a 90-m range with 102 monitoring points, each exhibiting a sensitivity of 63 ± 5 nTHz^–1/2^. The sensor also maintained stability in underwater tests (Fig. 9o, p). This fiber is expected to drive new applications for non-contact remote sensing in ferrous metal detection and biomedical fields.

Humidity, representing the amount of water vapor in the air, is a critical environmental parameter. Humidity sensors typically measure relative humidity (RH) and are widely used in environmental monitoring, industrial production, and medical fields [172–174]. However, the challenge of integrating thermal drawing processes to achieve flexible humidity sensors with both scalability and excellent sensing performance has persisted. So far, only one capacitive humidity fiber sensor has been fabricated. Tousi et al. [53] selected porous PEI as the sensing material, copper wires as the electrodes, and PPSU as the cladding to fabricate the preform (Fig. 9q). After drawing, the cladding was physically removed. The humidity tests were conducted in the device shown in Fig. 9r. The polymer's dielectric constant changed linearly with increasing moisture absorption, but its sensitivity was relatively low. To enhance performance, by controlling the water content of the preform and the cooling rate during the thermal drawing process, they fabricated an in situ porous structure in the PEI. Combined with capillary condensation phenomena [175], the sensor achieved a sensitivity of 0.39 pF/% RH at higher RH levels (70%–80%) (Fig. 9s). Additionally, the sensor exhibited low-temperature dependency and excellent cycling stability. In the future, further improvements in various performance of humidity sensors are expected.

### Multifunctional Sensors

With the development of flexible electronics, improving integration and expanding functionality diversity have become key trends in the future. Researches on highly integrated and highly sensitive multiresponse sensors are becoming increasingly urgent [176]. Currently, various methods, such as wet spinning, chemical vapor deposition, and spin coating, have been applied in multifunctional sensors [177–179]. The thermal drawing process can produce nanometer-scale fibers with complex geometries and integrated multiple functions, making it a promising multifunctional sensing process [45]. However, at present, thermally drawn fiber-based multifunctional sensors are still in their infancy, with only a few studies conducted.

**Fig. 10:**
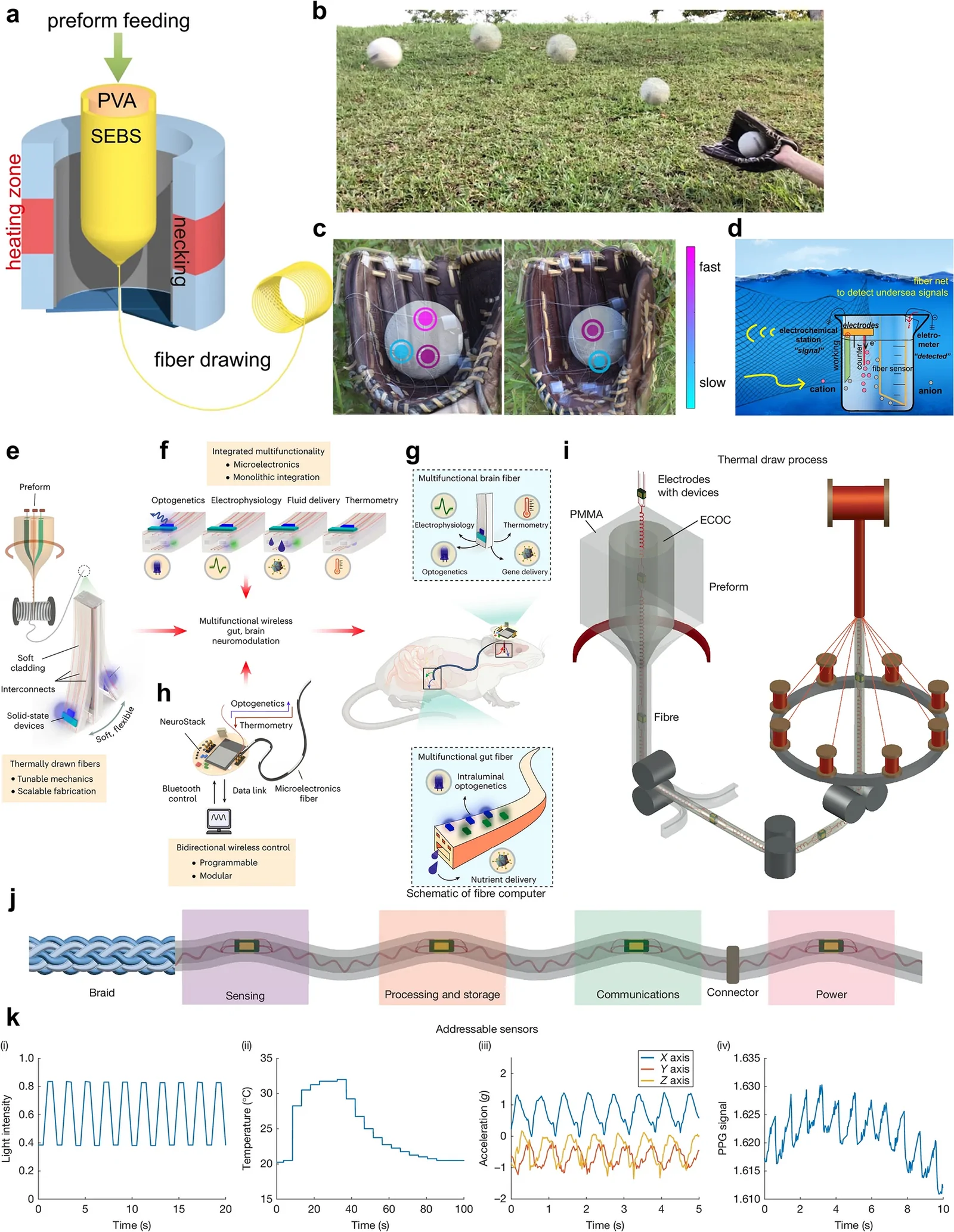
Thermally drawn fibers for multifunctional sensing.** a** Fabrication of a mechanical and ion concentration sensor. **b, c** Application as a mechanical sensor. **d** Fiber for ion movement detection. Reproduced with permission [180] Copyright 2021 Springer. **e**–**h** Images of microelectronics-integrated multifunctional fiber that enable temperature and biological sensing. Reproduced with permission [76] Copyright 2024 Springer. **i** Fabrication of a fiber computer.** j** The image of a fiber computer incorporating diverse embedded devices.** k** Characteristic time series plots obtained from four sensors. Reproduced with permission [181] Copyright 2025 Springer

Chen et al. [180] developed a multifunctional fiber sensor based on TENG and electrochemical principles that can monitor contact positions and changes in ion concentration. They used a soluble-core method to fabricate hollow SEBS fibers (Fig. 10a), followed by the infusion of liquid metal electrodes into the hollow fibers. This approach circumvented the viscosity limitations imposed by thermal drawing, thereby expanding the range of material choices. This fiber, with superior flexibility and stretchability, can be applied on complex surfaces. For example, when integrated into a baseball glove, the fiber sensing network can three-dimensionally locate contact positions and estimate contact speed based on the magnitude of electrical signals (Fig. 10b, c). The fiber can also be integrated into a three-electrode electrochemical system as a conductive pathway (as shown in Fig. 10d). By applying an external voltage, the ion migration in the solution lead to changes in the internal current of the fiber. Combining its waterproof and easily encapsulated characteristics, the fiber can be utilized for undersea detection to sense changes in ion concentration.

Sahasrabudhe et al. [76] integrated multiple μLEDs, electrodes, and microfluidic channels to fabricate a neural probe that can detect temperature as well as transmit and record physiological signals (Fig. 10e–h). The μLEDs were key components of the probe, enabling easy temperature sensing due to the linear temperature dependence of resistance. They also served as light sources to apply optogenetic stimulation. The probe was first applied to the complex-shaped intestine. Furthermore, by coupling with the NeuroStack module, the probe enabled real-time wireless control of sensory epithelial cells and data transmission, overcoming several limitations of wireless bioelectronics. This probe provides crucial information for establishing bidirectional communication between organs such as the intestines and the brain.

Recently, Gupta et al. [181] have fabricated a single-fiber computer integrating multiple sensing, storage, processing, and communication functions. The fiber used a foldable interposer method to map the two-dimensional layout of planar microdevices to a three-dimensional fiber structure, combined with helical copper wires and an elastic polymer cladding (Fig. 10i), endowing the fiber with a stretchability of over 60% and washability. Each fiber integrated a 32-bit floating-point microcontroller (MCU) and four multimodal sensors (a photodetector, a temperature sensor, a photoplethysmography (PPG) sensor, and an accelerometer) (Fig. 10j, k) and achieved distributed data processing via the I2C bus. Based on a wireless communication scheme, embedding four fibers into the clothing of the four limbs can realize physical activity prediction with a probability of 95%. This fully demonstrates the advantages of a distributed multifunctional sensing network.

In general, leveraging the unique advantages of thermal drawing process to integrate multiple commercial microsensors into fibers represents an effective approach to achieving multifunctional sensing. In the future, with the integration of the "Moore's law for fibers" [43], there will be a greater variety, higher density, and enhanced stability of microdevices that can be integrated into fibers. Consequently, thermally drawn fiber-based multifunctional sensors will become more ubiquitous.

## Conclusions and Outlook

In recent years, thermally drawn fiber sensors have advanced rapidly. TDFSs integrate a variety of materials, including metals, semiconductors, and polymers, and cover multiple sensing principles such as resistance, capacitance, piezoelectricity, triboelectricity, photoelectricity, and thermoelectricity. These sensors achieve a wide range of sensing functions for temperature, acoustic, mechanical, chemical, biological, optoelectronic, flow, magnetic, and humidity sensing. Here, we summarize some challenges that TDFSs still faces and the potential development directions in the future.

*Material*: Recently, the variety of materials integrated into TDFSs has been increasing. Liquid metal electrodes, which are more deformable, have shown great compatibility with the thermal drawing process and have been widely studied. Furthermore, with the introduction of rheological criteria, many of TPEs have emerged as viable options for function materials and cladding. Additionally, innovative methods have been developed to draw materials previously incompatible with the thermal drawing process. Therefore, it is urgent to establish a comprehensive material library that compiles various parameters of materials meeting the selection criteria. This would facilitate the initial material selection for researchers and accelerate the research and development cycle.

*Structure*: Fiber structure determines its applications. Over the past decades, to enhance performance, researchers have made multiple improvements to the structure. The preform structure has evolved from basic rectangular/circular structures [113] to multilayer folding structure [101, 162], and further to "Swiss roll" structure [56, 72]. These advancements have significantly increased the contact area and improved the performance of TDFSs. From the perspective of fiber microstructure, the emergence of techniques such as surface textures [127], direct imprinting technology [41], and double-helical structures of liquid metal electrodes [79] represents the development of thermally drawn fibers toward diversified structures and functionalities. However, currently, there is limited understanding of the microstructure-forming mechanism of thermally drawn fibers and the relationship between microstructure and functionalities. Therefore, it is necessary to focus on the microstructure and intensify research on the microstructure-forming mechanism of TDFSs to provide a foundation for performance enhancement.

*Fabrication*: The thermal drawing process exhibits excellent technological compatibility. It has been successfully integrated with techniques such as lithography and 3D printing. Additionally, multiple drawing steps can effectively reduce fiber sizes to meet application requirements. However, in actual fabrication, fractures and cracks remain the most challenging issues to address. Proper material selection and structure can mitigate these problems to a certain extent. Furthermore, various post-processing techniques are also meant to reduce residual stresses within fibers. Looking ahead, further diversification of post-processing techniques could pave the way for stable large-scale production of high-performance TDFSs.

*Function*: Currently, although TDFSs achieve diverse functions, most are concentrated in the single-sensing field. With the advancement of flexible electronics, high integration and miniaturization of devices are certainly the trends. The research on composite sensors will surely be a crucial part of the trends. Current sensors often struggle to detect multiple stimuli simultaneously. Integrating microelectronic devices into fibers is one possible approach. However, in order to simplify structure and reduce size, decoupling multiple response signals within composite sensors remains a pressing challenge to be solved.

*Stability*: For sensors applied in flexible smart textiles, the long-term stability and chemical stability of the materials are critical factors. However, most researchers have not thoroughly investigated these aspects. Therefore, it is imperative to conduct in-depth analyses of failure mechanisms in TDFSs under complex environments after prolonged operation, ensuring the long-term mechanical stability and chemical durability of TDFSs.

As a prominent branch of flexible electronics and intelligent sensing, TDFSs will see substantial improvements in material diversity, structural customization, process stability, and functional integration. Moreover, in the context of interdisciplinary development, the deep integration of large-scale fabricated TDFSs with flexible e-skin and smart textiles will revolutionize the design logic of human–machine interaction interfaces. When synergized with deep learning algorithms, this convergence will enable more accurate and convenient recognition and prediction of diverse human physiological signals. TDFSs represent a highly promising class of flexible sensors, destined to become seamlessly integrated into daily life and propel technological innovations across diverse fields such as intelligent robotics and biomedical engineering.
